# Comparative transcriptomic analysis of gill reveals genes belonging to mTORC1 signaling pathway associated with the resistance trait of shrimp to VP_AHPND_

**DOI:** 10.3389/fimmu.2023.1150628

**Published:** 2023-04-18

**Authors:** Yuan Liu, Yang Yu, Shihao Li, Mingzhe Sun, Fuhua Li

**Affiliations:** ^1^ Chinese Academy of Sciences (CAS) and Shandong Province Key Laboratory of Experimental Marine Biology, Institute of Oceanology, Chinese Academy of Sciences, Qingdao, China; ^2^ Laboratory for Marine Biology and Biotechnology, Qingdao National Laboratory for Marine Science and Technology, Qingdao, China; ^3^ Center for Ocean Mega-Science, Chinese Academy of Sciences, Qingdao, China

**Keywords:** *Litopenaeus vannamei*, RNA-seq, hepatopancreatic necrosis disease, *Vibrio parahaemolyticus*, disease resistance

## Abstract

Selective breeding for acute hepatopancreatic necrosis disease (AHPND) resistant shrimp is an effective way to deal with heavy losses to shrimp aquaculture caused by AHPND. However, knowledge about the molecular mechanism of susceptibility or resistance to AHPND is very limited. We herein performed a comparative transcriptomic analysis of gill tissue between AHPND susceptible and resistant families of the white Pacific shrimp *Litopenaeus vannamei* during *Vibrio parahaemolyticus* (VP_AHPND_) infection. A total of 5,013 genes that were differentially expressed between the two families at 0 and 6 h post-infection, and 1,124 DEGs were shared for both two time points. Both GO and KEGG analyses in each or two time point’s comparisons showed DEGs involved in endocytosis, protein synthesis and cell inflammation were significantly enriched. Several immune DEGs including PRRs, antioxidants and AMPs were also identified. The susceptible shrimp showed enhanced endocytosis, higher aminoacyl-tRNA ligase activity and occurrence of inflammatory response, while the resistant shrimp had much more strong ability in ribosome biogenesis, antioxidant activity and pathogen recognition and clearance. These genes and processes were mostly associated with mTORC1 signaling pathway, which could reflect differences in cell growth, metabolism and immune response between the two families. Our findings indicate a close link between mTORC1 signaling-related genes and *Vibrio*-resistance phenotype of shrimp, and provide new clues for further research on resistance strategy of shrimp to AHPND.

## Introduction

The Pacific white shrimp *Litopenaeus vannamei*, accounting for more than 80% of total shrimp production, is the dominant crustacean species in aquaculture worldwide (1). China is the world’s largest producer of *L. vannamei* and its production reached 1.98 million tons in 2021 (2). However, with the rapid development of shrimp aquaculture industry, *L. vannamei* farming have encountered tremendous challenges caused by disease outbreaks. Acute hepatopancreatic necrosis disease (AHPND) or early mortality syndrome (EMS) caused by bacteria has devastating effects on the global shrimp farming since its first outbreak in China in 2009 (3).

Many efforts have been done to elucidate the pathology and physiology of AHPND-infected shrimp. AHPND is known to be mainly caused by *Vibrio parahaemolyticus* carrying the *pirA* and *pirB* toxin genes in its plasmid (VP_AHPND_) (4). VP_AHPND_ may initially colonize in the shrimp stomach and cause cellular damage by releasing virulent toxin into the shrimp hepatopancreas (5, 6). The characteristic symptoms of AHPND are pale and atrophied hepatopancreas, accompanied by sloughing of hepatopancreas tubule epithelial cells in the early stage, and necrosis of tubule epithelial cells and inflammatory responses in the late stages (6, 7). Previous studies on the immune response of *L. vannamei* to VP_AHPND_ infection have focused on the VP_AHPND_ target tissues including hepatopancreas and hemocytes (8–12). However, it is still largely unknown about how other shrimp tissues respond to VP_AHPND_ under the natural course of infection.

As a main site of interaction with the environment, the crustacean gill plays important roles not only in respiration and osmoregulation but also in immune defense (13, 14). For example, during foreign particle injection, hemocyte nodules could form in the gill (15). The gill is the site of accumulation of viable bacteria or their degradation products during infection (16). Previous transcriptomic studies examined the gill response of *L. vannamei* against WSSV and revealed that WSSV changed the expression of metabolic, immune, apoptotic and cytoskeletal genes in gills (17). All these studies suggest that the crustacean gill is more susceptible to pathogens and its immune response to VP_AHPND_ infection need to be further explored.

Screening and breeding of disease-resistant broodstock is an effective and sustainable way to control disease. Recently, several efforts have been devoted to examine the gene expression profiles between AHPND susceptible and resistant lines by RT-PCR (18) or transcriptome sequencing (19). However, the molecular mechanism, key genes and regulatory network underlying differences in AHPND tolerance between lines or families are still unclear. Therefore, our study aimed to compare the transcriptional responses in gill between AHPND susceptible and resistant *L. vannamei* during VP_AHPND_ infection. Several differentially expressed genes (DEGs) and the interactions of their pathways related to the susceptibility or resistance of shrimp against AHPND were identified. These findings will expand our knowledge about the molecular basis of AHPND susceptibility or resistance in *L. vannamei* and provide theoretical basis for disease-resistant shrimp breeding.

## Materials and methods

### Selection of susceptible and resistant families against AHPND

AHPND susceptible and resistant families of *L. vannamei* were selected and assessed according to methods described by Zhang et al. (19). In 2019, 79 full-sib families with an average body weight of 2.94 g were selected as the on-going family lines for evaluation of AHPND resistance. About 100 healthy shrimp from each family were subjected to the immersion challenge with the concentration of VP_AHPND_ as 1×10^7^ CFU/ml. The mortality of each family was recorded for 72 h. Considering the survival rate, growth stage and body weight, one susceptible family (S4383, body weight 3.68 ± 0.58 g) and one resistant family (R4345, body weight 3.45 ± 0.44 g) were selected for further study.

### Sample collection

To compare molecular immune mechanism in the AHPND susceptible and resistant families, a second immersion challenge with the concentration of VP_AHPND_ as 5×10^6^ CFU/ml was conducted. Due to the immersion challenge, gill samples of nine individuals were randomly collected at the time point of 0, 6, 12 and 24 hours post-infection (hpi) from S4383 and R4345 families. Gills from three individuals were mixed together as one sample, and each family contained three samples at each time point. All gill samples were rapidly frozen in liquid nitrogen and stored at −80°C for further bacterial load detection and transcriptomes sequencing.

### VP_AHPND_ load detection

A TaqMan probe-based fluorescence real time PCR method was established to detect the load of *V. parahaemolyticus* in shrimp according to the previous assay with some modifications (20). Specific primers and probe were designed based on the sequence of *PirA^Vp^* in *V. parahaemolyticus*. Gene specific primers PirA-F (5′-CGGAAGTCGGTCGTAGTGTA-3′) and PirA-R (5′-TGTGATTTAGCCACTTTCCAG-3′) was used to amplify a product of 112 bp *PirA^Vp^*. The TaqMan probe (5′-CCGCCAGCCATAAATGGCGCACC-3′) were synthesized and labeled with 6-carboxyfluorescein (FAM) at the 5′end and Black Hole Quencher 1 (BHQ1) at the 3′end.

The 112-bp target fragment was cloned into pUC57 vector and transformed into the competent cells of *Escherichia coli* DH5α. After confirmed by sequencing, the positive plasmid was extracted by Plasmid DNA Mini Kit (OMEGA Bio-tek, USA). The concentration of the plasmid was then determined using a NanoDrop 2000 (Thermo Fisher Scientific, USA). The copy number of the plasmid containing the 112-bp insert was estimated, and a series of dilutions were prepared as standards. The standard curve was generated based on the 10-fold serial dilutions of *PirA^Vp^* positive plasmid (10^2^ to 10^10^ copies/μL) used as templates for the TaqMan qPCR assay.

The TaqMan reactions were performed in a 10-μL reaction system consisting of 5 μL 2×AceQ Universal U^+^ Probe Master Mix V2 (Vazyme, China), 10 μM of each primer (PirA-F/R), 10 μM TaqMan probe, 1 μL DNA template and 3.5 μL sterile distilled H_2_O. The PCR reactions carried out in an Eppendorf Mastercycler ep realplex real-time PCR system (Eppendorf, Germany). The PCR program was 37°C for 2 min, 95°C for 5 min, followed by 45 cycles of 95°C for 10 s and 60°C for 35 s. The load of *V. parahaemolyticus* in gill samples of S4383 and R4345 families were determined by the TaqMan qPCR assay. Genomic DNA was extracted from gill samples using Plant Genomic DNA Kit (TianGen, Beijing, China). Copy number of PirA^Vp^ per ng DNA of different sampling points from S4383 and R4345 families was calculated based on the standard curve.

### RNA-seq of gill

Gill samples collected at 0 and 6 h post-infection of S4383 and R4345 families were used for transcriptome sequencing. Total RNA was extracted from gill samples using Trizol reagent kit (Invitrogen, Carlsbad, CA, USA) according to the manufacturer’s protocol. RNA quality and integrality were determined on an Agilent 2100 Bioanalyzer (Agilent Technologies, USA). The mRNA was isolated from total RNA by Oligo (dT) beads and fragmented into short fragments using fragmentation buffer. First cDNA was reverse-transcribed, second-strand cDNA were synthesized and 3′-ends were repaired and poly (A) added. Sequencing adapters were then ligated to cDNA fragments, and the libraries were sequenced using Illumina HiSeq2500 by Gene Denovo Biotechnology Co., Ltd. (Guangzhou, China).

### Sequence assembly and functional annotation

The raw data were trimmed and filtered to remove adapters, reads with unknown nucleotides (N) and low-quality reads (Q ≤ 20) by fastp version 0.18.0 (21). The clean reads were mapped to reference genome (22) by TopHat2 version 2.0.3.12 (23). The reconstruction of transcripts was carried out with software Cufflinks (24). All reconstructed transcripts were aligned to reference genome, and novel genes were annotated by BLAST against NCBI nonredundant (Nr), Swiss-Prot, KEGG and GO databases.

### Differential expression analysis

Gene expression levels were calculated with fragments per kilobase of million mapped reads (FPKM) values and differentially expressed genes (DEGs) were identified by DESeq2 software (25). Genes were considered as DEGs based on the FPKM values with the parameter of false discovery rate (FDR) below 0.05 and absolute value of log2 fold change (FC) above 1. GO function and KEGG pathway enrichment analyses were used to categorize DEGs and evaluate DEGs in the potential biological pathways. Based on public databases and the published literatures, the crucial DEGs related to immunity were manually checked.

### Validation of DEGs by quantitative real-time PCR

To validate RNA-seq data and expression profiles obtained from DESeq analysis, 15 DEGs were selected to evaluate the transcriptome sequencing result by RT-qPCR. The gene-specific primers designed for the 16 genes are listed in **Supplementary Table S1**.

About 1 μg of total RNA was used to synthesize cDNA by PrimeScript RT reagent Kit with gDNA Eraser kit (Takara, Japan) according to the manufacturer’s instructions. The qRT-PCR reaction was performed in 10 µL reaction system, containing 2.28 µL of sterile distilled H_2_O, 3.33 µL of 2×SYBR Premix Ex Taq (TaKaRa), 0.13 µL of 50× ROX Reference Dye, 0.13 µL of each primer (10 µmol L^−1^) and 4 µL of the diluted cDNA. The PCR program was 95°C for 5 min, followed by 40 cycles of 95°C for 5 s and 60°C for 31 s. Each sample was run in triplicate. The relative expression level was calculated by 2^-ΔΔCt^ method using 18S rRNA as internal standardization (26). Data were analyzed *via* one-way ANOVA using SPSS 16.0, and the difference was considered significant if *P* values less than 0.05 and 0.01.

### Validation of candidate genes in other families

In order to validate the identified DEGs, other susceptible family (S4301, body weight 4.58 ± 0.70 g) and resistant family (R4347, 3.73± 0.36 g) were collected, and the DEGs were further validated in these two families by qRT-PCR. The designed gene-specific primers were listed in **Supplementary Table S1**. The qRT-PCR experiment and data analysis were performed as described in Validation of DEGs by quantitative real-time PCR.

## Results

### Assessing AHPND susceptible and resistant families of *Litopenaeus vannamei*

The survival rates of the tested 79 families post VP_AHPND_ immersion challenge were presented in **Supplementary Figure S1**. 4383 family with the lowest survival rate (1.14%) and 4345 family with the higher survival rate (79.75%) were selected as the susceptible family (S4383) and the resistant family (R4345) for further study, respectively.

**Figure 1:**
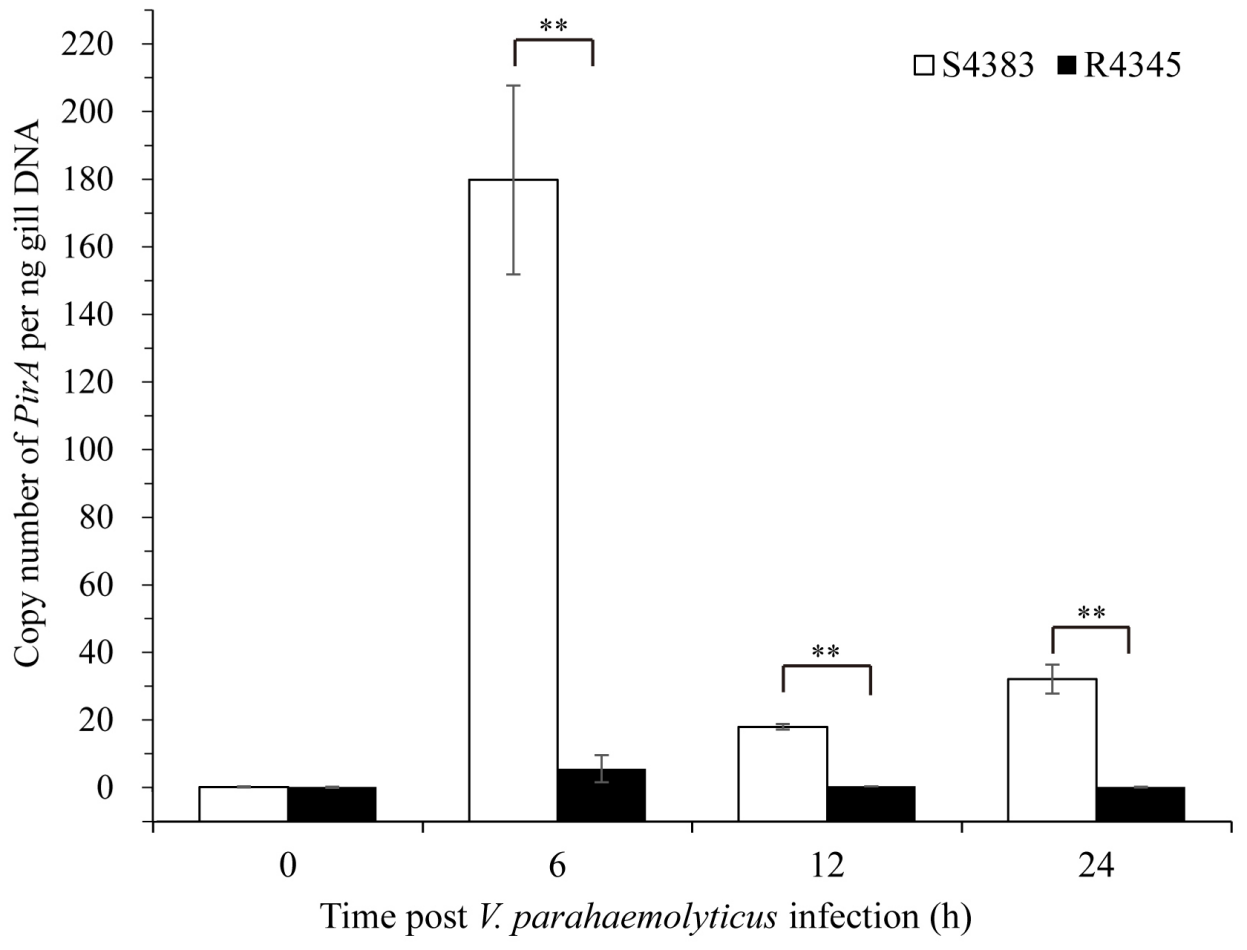
Copy number of *PirA^Vp^* for gills from VP_AHPND_-infected shrimp of S4383 and R4345 families. Data are represented as mean ± S.D. (n = 3). Significant differences between S4383 and R4345 at the same sampling point are indicated with two asterisks at *P* < 0.01.

A TaqMan probe selected from *PirA^Vp^* of *V. parahaemolyticus* was used to detect the VP_AHPND_ load in shrimp. No copy number of *PirA^Vp^* was found in the gills of non-infected shrimp of S4383 and R4345 families, while during VP_AHPND_ infection, R4345 family showed significantly lower copy numbers of *PirA^Vp^* in the gills as compared to S4383 family (**Figure 1**). The highest VP_AHPND_ load was detected in the gills of S4383 family at 6 h post-infection (179.63 *PirA^Vp^* copies/ng DNA), which was over 30 times higher than that in R4345 family (**Figure 1**).

### Transcriptome sequencing data

A summary of the transcriptome sequencing of *L. vannamei* was shown in **Supplementary Table S2**. The raw reads in each library ranging from 47,695,886 to 78,516,062 were obtained and submitted to Sequence Read Archive (SRA) in NCBI with the accession numbers SRR22936311-SRR22936318. After removing and filtering adapter, poly-N and low quality reads, a total of 458,042,932 clean reads were retained, in which 137,009,336 reads for S4383 at 0 hpi (S4383-0-G), 101,482,088 reads for S4383 at 6 hpi (S4383-6-G), 121,736,918 reads for R4345 at 0 hpi (R4345-0-G) and 97,814,590 reads for R4345 at 6 hpi (R4345-6-G). An average of 85.04% clean reads were mapped to the reference *Litopenaeus vannamei* genome, and approximately 18,991 genes were detected in each sample. The overall expression levels in the biological replicates of each group were highly similar to each other (Pearson’s r > 0.93; **Supplementary Figure S2**), illustrating that our RNA-seq data was of suitable quality for transcriptome analysis.

### Identification of differentially expressed genes in each comparison

**Figure 2:**
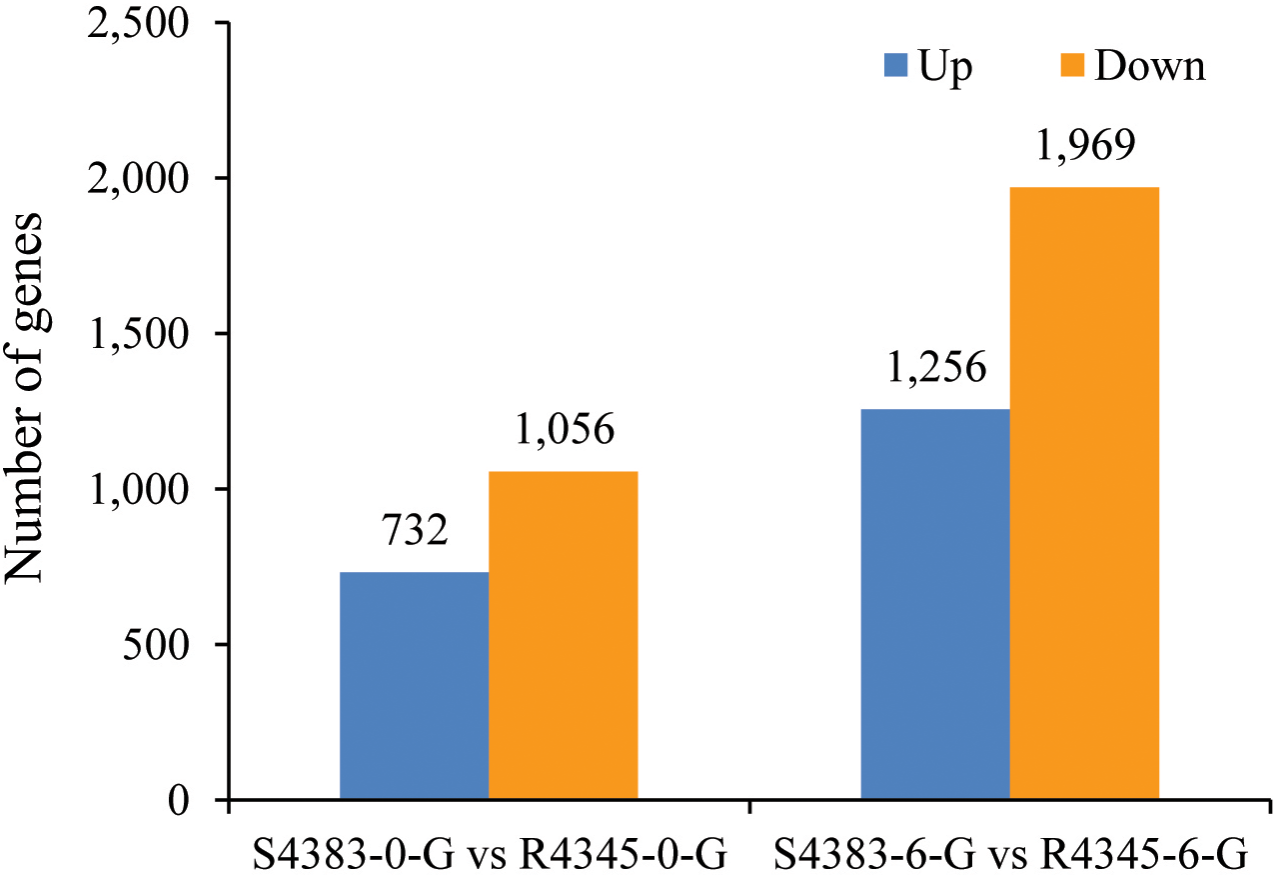
The amount of differentially expressed genes (DEGs) between S4383 family and R4345 family during VP_AHPND_ infection. Up represents highly expressed genes in R4345. Down represents highly expressed genes in S4383.

A total of 5,013 genes were identified as DEGs between S4383 and R4345 during VP_AHPND_ infection (**Figure 2**). Compared to S4383-0-G group, 1,788 DEGs were detected in the R4345-0-G group, of which 732 DEGs were up-regulated and 1,056 DEGs were down-regulated. In the R4345-6-G group, a total of 3,225 DEGs were identified, including 1,256 up-regulated and 1,969 down-regulated genes (**Figure 2**). To verify the result of RNA-seq analysis, seven DGEs were selected for qRT-PCR to further investigate the expression profiles. The results showed that all of them were consistent with the transcriptome data (**Supplementary Figure S3**).

**Figure 3:**
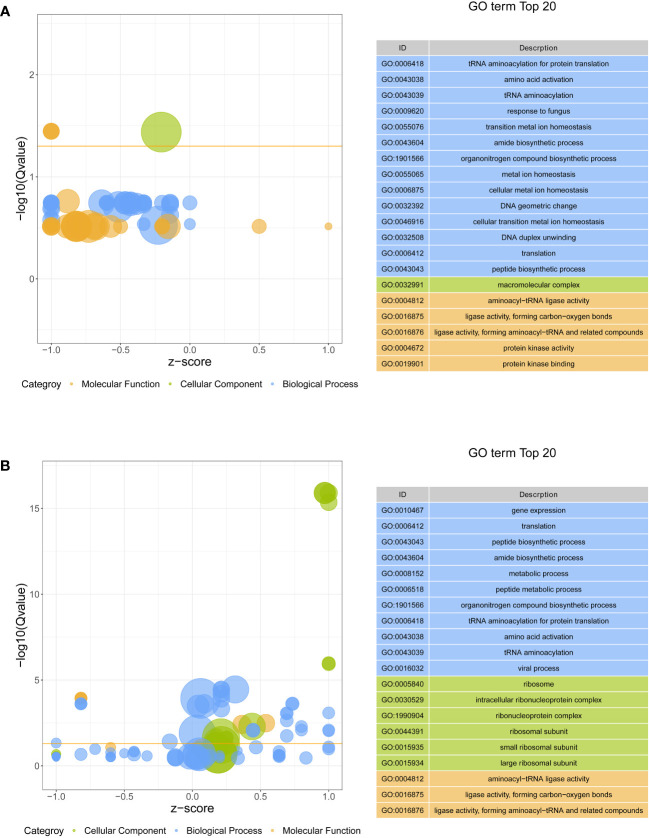
Gene ontology (GO) enrichment analysis of DEGs in each comparison group. **(A)** S4383-0-G vs R4345-0-G, **(B)** S4383-6-G vs R4345-6-G. Bubble plot showing the top 20 enriched GO terms in biological process (blue), molecular function (orange) and cell component (green). The y-axis belongs to -log10 (Q value), and the x-axis belongs to z-score. The horizontal line indicates the significance threshold (Q value=0.05).

All DEGs were performed on GO function and KEGG pathway enrichment analysis. In GO analysis, two GO terms, macromolecular complex (GO:0032991) in cellular component and aminoacyl-tRNA ligase activity (GO:0004812) in molecular function, were significantly enriched in S4383-0-G vs R4345-0-G (**Figure 3A**, **Supplementary Table S3**). Within macromolecular complex, the most upregulated DEGs in the R4345-0-G group were ribosomal proteins and regulators of translation, while in the S4383-0-G group, the upregulated DEGs were mainly related to the processes of cell growth and death including DNA replication, mitosis and cellular organelles (**Supplementary Figure S4**). In S4383-6-G vs R4345-6-G, ribosome (GO:0005840), intracellular ribonucleoprotein complex (GO:0030529), ribonucleoprotein complex (GO:1990904), ribosomal subunit (GO:0044391) and small ribosomal subunit (GO:0015935) in cellular component were the top five enriched GO terms (**Figure 3**). And the DEGs involved in ribosome were significantly upregulated in the R4345-6-G group (**Supplementary Figure S5**).

**Figure 4:**
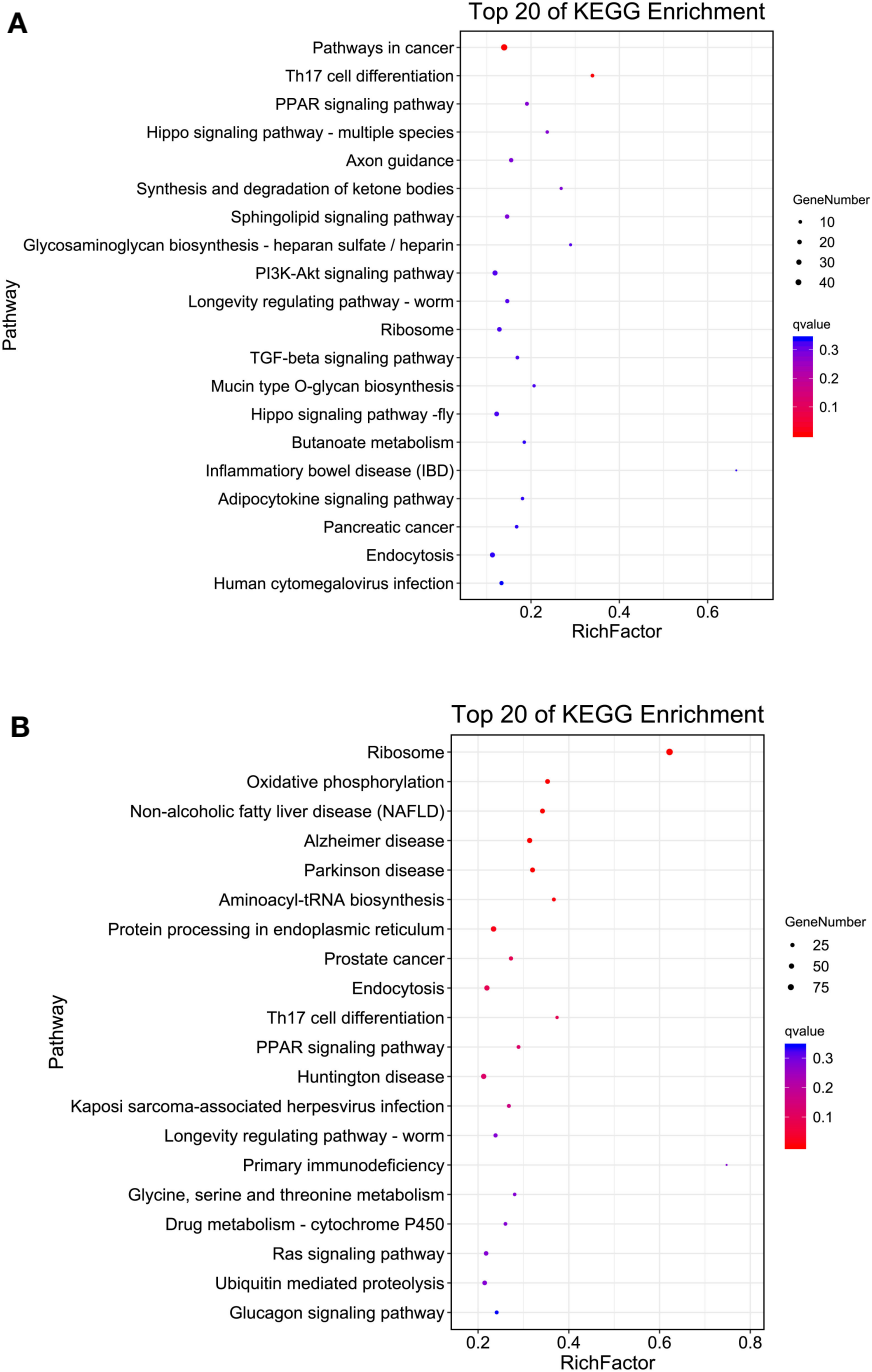
KEGG pathway enrichment analysis of DEGs in each comparison group. **(A)** S4383-0-G vs R4345-0-G, **(B)** S4383-6-G vs R4345-6-G. The y-axis belongs to specific pathway, and the x-axis belongs to enrichment factor. The size and colors of the dots represent the number of genes and q values, respectively (The dots with larger-size indicated a higher number of genes in the pathway).

KEGG enrichment analysis showed that pathways in cancer (ko05200), Th17 cell differentiation (ko04659), PPAR signaling pathway (ko03320), Hippo signaling pathway (ko04392) and axon guidance (ko04360) were the top five enriched KEGG pathways in S4383-0-G vs R4345-0-G (**Figure 4A**, **Supplementary Table S4**). In S4383-6-G vs R4345-6-G, the pathway predominantly enriched for DEGs were ribosome (ko03010), oxidative phosphorylation (ko00190) and three disease-related pathways (**Figure 4B**). There were 39 DEGs with 36 KO terms in oxidative phosphorylation, including 34 up-regulated in the R4345-6-G group and two up-regulated in the S4383-6-G (**Supplementary Figure S6**). Among the top 20 enriched pathways, endocytosis and ribosome were well represented in both S4383-0-G vs R4345-0-G and S4383-6-G vs R4345-6-G comparison groups. The most upregulated DEGs involved in endocytosis were in S4383-0-G and S4383-6-G groups, while in ribosome, all related DEGs were upregulated in R4345-0-G and R4345-6-G groups (**Supplementary Figures S7**, **S8**).

### Differentially expressed genes shared in the two comparisons

**Figure 5:**
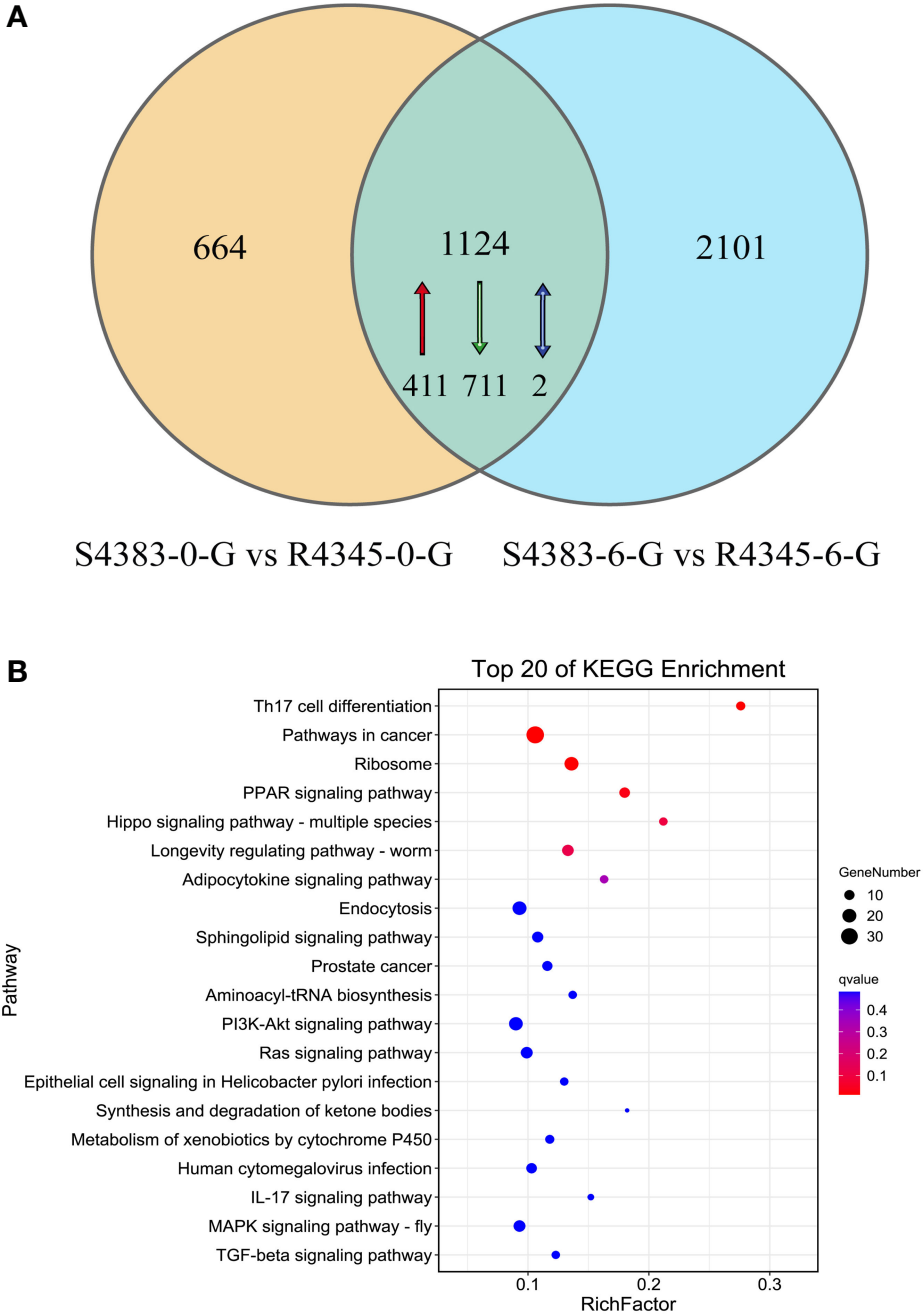
DEGs shared between the two comparison groups S4383-0-G vs R4345-0-G and S4383-6-G vs R4345-6-G. **(A)** Venn diagram showing the numbers of the shared DEGs. Upregulated genes in R4345 are marker with red arrow, down regulated genes in R4345 are marked with green arrows, and the opposite expression trend in R4345 at 0 and 6 h post-infection are marked with blue arrows. **(B)** KEGG pathway enrichment analysis of the shared DEGs. The y-axis belongs to specific pathway, and the x-axis belongs to enrichment factor. The size and colors of the dots represent the number of genes and q values, respectively (The dots with larger-size indicated a higher number of genes in the pathway).

A Venn diagram analysis showed that 1,124 DEGs were shared between the two comparison groups S4383-0-G vs R4345-0-G and S4383-6-G vs R4345-6-G. (**Figure 5**, **Supplementary Table S5**). Of these, 411 DEGs were upregulated in the R4345 group, 711 DEGs were downregulated in the R4345 group, and 2 DEGs showed opposite expression trend in R4345 at 0 and 6 hpi (**Table S3**). There were 195 DEGs were involved in GO classifications and the major enriched GO terms were related to aminoacyl-tRNA ligase activity (GO:0004812) in molecular function, and cellular transition metal ion homeostasis (GO:0046916) and leukocyte homeostasis (GO:0001776) in biological process (**Supplementary Table S6**).

KEGG analysis revealed that 351 DEGs with KO terms were involved in the predicated pathways. The most abundant pathways included Th17 cell differentiation (ko04659), pathways in cancer (ko05200), ribosome (ko03010), PPAR signaling pathway (ko03320) and Hippo signaling pathway (ko04392) (**Figure 5B**). Among the top 20 ranked KEGG pathways, five pathways, including Th17 cell differentiation (ko04659), ribosome (ko03010), PPAR signaling pathway (ko03320), longevity regulating pathways (ko04212) and endocytosis (ko04144), were shared in each comparison group (**Figure 4**) and the two comparison groups (**Figure 5B**).

**Figure 6:**
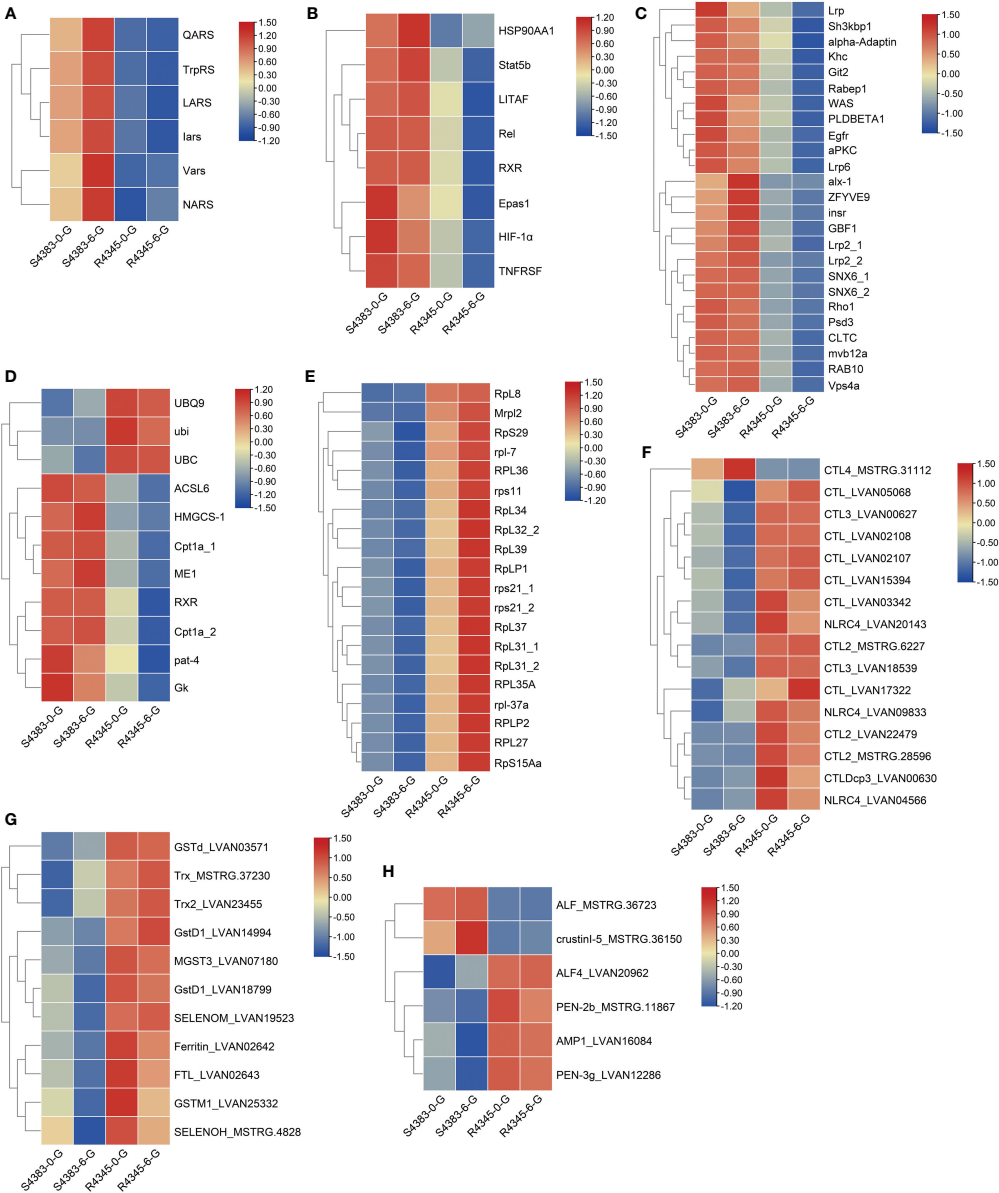
Heatmap of the expression patterns of key DEGs shared between the two comparison groups S4383-0-G vs R4345-0-G and S4383-6-G vs R4345-6-G. **(A)** DEGs involved in aminoacyl-tRNA ligase activity (GO:0004812). **(B)** DEGs involved in Th17 cell differentiation (ko04659) and inflammation. **(C)** DEGs involved in endocytosis (ko04144). **(D)** DEGs involved in PPAR signaling pathway (ko03320). **(E)** DEGs involved in ribosome (ko03010). **(F)** DEGs identified as pattern recognition receptors (PRRs). **(G)** DEGs identified as antioxidants. **(H)** DEGs identified as antimicrobial peptides (AMPs).

Of the aminoacyl-tRNA ligase activity, six aminoacyl-tRNA synthetases/ligases, including glutamine-tRNA ligase (QARS), tryptophan-tRNA ligase (TrpRS), leucine-tRNA ligase (LARS), isoleucyl-tRNA synthetase (Iars), valine-tRNA ligase (Vars) and asparagine-tRNA ligase (NARS), were highly expressed in S4383-0-G and S4383-6-G groups (**Figure 6A**). LARS is reported as a leucine sensor for mechanistic target of rapamycin complex 1 (mTORC1) signaling, and the expression pattern of mTORC1-related genes were further examined. The results showed that mLST8, the essential constituent of mTORC1, and RPS6KB1, the key downstream target of mTORC1, were differentially expressed (**Supplementary Figure S9**).

Six DEGs related to Th17 cell differentiation (ko04659), including HSP90AA1, Stat5b, Relish, RXR and hypoxia inducible factor-1 (HIF-1α and Epas1), and two inflammation-related genes, including TNF receptor superfamily (TNFRSF) and lipopolysaccharide-induced TNF-a factor (LITAF), showed high expression in S4383-0-G and S4383-6-G groups (**Figure 6B**). In endocytosis (ko04144), a total of 25 DEGs involved in receptor-mediated endocytosis and clathrin dependent or independent endocytosis, were also highly expressed in the susceptible families (**Figure 6C**, **Supplementary Figure S10A**). The endocytosis related receptors and proteins, including insulin-like peptide receptor (Insr), epidermal growth factor receptor (Egfr), low-density lipoprotein receptor-related proteins (Lrp2 and Lrp6), clathrin heavy chain (CLTC) and flotillin-1 (Flo1), were upregulated in the susceptible families (**Figure 6C**). In PPAR signaling pathway (ko03320), three polyubiquitin proteins were highly expressed in R4345-0-G and R4345-6-G groups, while five DEGs involved in lipid metabolism and gluconeogenesis were highly expressed in S4383-0-G and S4383-6-G groups (**Figure 6D**, **Supplementary Figure S10B**). Consistent with that in each comparison group, a total of 20 DEGs related to ribosome (ko03010) were highly unregulated in the resistant family (**Figure 6E**).

To further compare the immune responses in the gills between the susceptible family and the resistant family, we identified immune-related DEGs based on their sequence annotation and functional classification. The immune-related DEGs could be divided into three categories, including pattern recognition receptors (PRRs), antioxidants and antimicrobial peptides (AMPs). Except CTL4, most PRRs showed high expression in R4345-0-G and R4345-6-G groups (**Figure 6F**). All identified antioxidant enzymes were highly expressed in R4345-0-G and R4345-6-G groups (**Figure 6G**). Four AMPs were also observed to be upregulated in the resistant family, while two transcripts encoding ALF and crustinI-5 were highly expressed in the susceptible family (**Figure 6H**).

### Verification of candidate genes in other families

**Figure 7:**
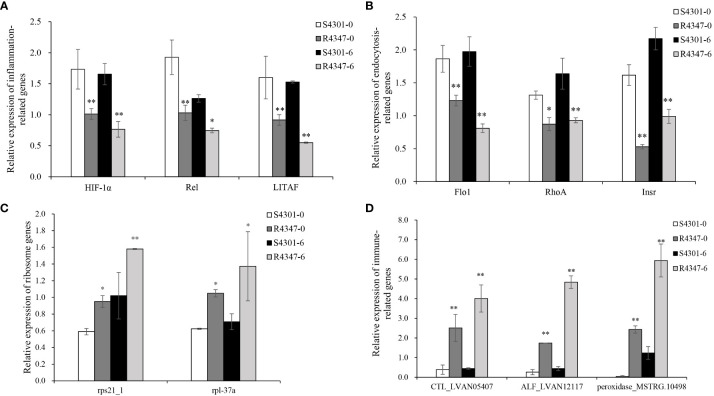
The relative expression of DEGs in the gills from the susceptible family S4301 and the resistant family R4347. **(A)** Inflammation-related genes. **(B)** Endocytosis-related genes. **(C)** Ribosome genes. **(D)** Immune-related genes. Vertical bars represent the mean ± S.E. (n = 3). Significant differences between R4347 and S4301 at the same sampling point are indicated with an asterisk at *P* < 0.05, and two asterisks at *P* < 0.01.

After being challenged by VP_AHPND_, the survival rate of family 4347 was over 2.5 times higher than that of family 4301 (68.97 vs. 24.71%). Considering the survival rate and growth stage, 4301 and 4347 families were selected as the susceptible family (S4301) and the resistant family (R4347) to validate the identified DEGs. Among the selected DEGs, 11 genes, including three inflammation-related genes, three endocytosis-related genes, two ribosome genes and three immune-related genes, were verified successfully in the gills of S4301 and R4347 (**Figure 7**). The expression levels of inflammation and endocytosis-related genes were significantly upregulated in S4301 groups, while ribosome and immune-related genes showed higher expression in R4347 groups.

## Discussion

Selective breeding for disease-resistant broodstock is an accurate, feasible and sustainable approach for controlling disease. In our previous work, we have carried out systematic family selection for the resistance trait to VP_AHPND_ in *L. vannamei* (19, 27). In this study, based on the obtained different resistance phenotype of shrimp families, we firstly detected the dynamic changes of pathogenic bacteria between susceptible and resistant families during VP_AHPND_ immersion challenge. The significant higher VP_AHPND_ load were found in the gills of susceptible family at 6, 12 and 24 h post-infection, suggesting the gills of susceptible family appear to be more susceptible to bacterial penetration than those of resistant family. To learn the different underlying mechanisms, we further performed RNA-seq analysis to compare the transcriptional profiles in gills between the susceptible and resistant families of *L. vannamei* during VP_AHPND_ infection. To our knowledge, this is the first transcriptomics report in gill to delineate the relationship between gene expression and AHPND resistance phenotypes of *L. vannamei*.

Both GO and KEGG analyses in each or two time point’s comparisons showed DEGs in endocytosis, protein synthesis and cell inflammation were significantly enriched, and immune-related DEGs including PRRs, antioxidants and AMPs were also identified. And some DEGs were verified by other susceptible and the resistant families. Below we highlighted key genes in these categories and their potential functions in the context of AHPND resistant mechanisms in shrimp.

### Enhanced endocytosis in the susceptible family

Endocytosis is essential for the acquisition of extracellular material and involved in many cellular functions, such as nutrient uptake, receptor signaling, membrane remodeling and cell migration (28). Endocytosis also provides pathways, such as macropinocytosis, clathrin-mediated endocytosis and lipid rafts and caveolae pathways, through which many bacteria or viruses gain entry into host cells (29, 30). In this study, DEGs involved in receptor-mediated, and clathrin dependent or independent endocytosis were significantly upregulated in the susceptible family at 0 and 6 h post-infection, suggesting the active transport of endocytosis in the susceptible family. Membrane lipid raft gene flotillins have been reported to be involved in the process of *V. alginolyticus* or WSSV infection in crustaceans (31, 32). The upregulated expression of flotillin-1 and clathrin heavy chain in the susceptible family might indicate that active endocytosis could provide a possible pathway of VP_AHPND_ entry into the gill cells of *L. vannamei*.

Cells internalize hormones, such as insulin and growth factors EGF, *via* receptor-mediated endocytosis (33). The higher expression of insulin-like peptide receptor and epidermal growth factor receptor in the susceptible family might suggest the differential regulation of cell growth and energy metabolism between the two families. Nutrient sensing and energy metabolism are coordinated by networks of signaling cascades, for example, both insulin and EGF could activate mechanistic target of rapamycin Complex 1 (mTORC1) *via* PI3K/Akt signaling (34, 35). mTORC1 is a major signaling hub that integrates different inputs, such as nutrients, oxygen, energy, and growth factors, to regulate the metabolic pathways controlling cell growth and metabolism (36). Together with the following analyses in protein synthesis and cell inflammation, we suggest that mTORC1 related signaling pathways may play important roles in the resistance trait to VP_AHPND_ in *L. vannamei*.

### Differential expression in protein synthesis between the two families

In the present study, two categories aminoacyl-tRNA ligase activity and ribosome showing different expression patterns in the two families were mainly related to protein synthesis. Aminoacyl-tRNA synthetases (ARSs), also called aminoacyl-tRNA ligases, are enzymes that catalyze the ligation of tRNAs with their cognate amino acids and play an essential role in the initial steps of protein synthesis (37). Apart from this classical function, ARSs are also known to be involved in several metabolic and signaling pathways that are important for cell viability (38). Especially, ARSs could act as intracellular amino acid sensors to regulate signaling cascades that govern diverse cellular physiologies (37, 39). The higher expression of ARSs in the gills of the susceptible family, such as glutamine-tRNA ligase (QARS), leucine-tRNA ligase (LARS) and asparagine-tRNA ligase (NARS), suggest the changes in the intracellular levels of their cognate amino acids in the susceptible family. Previous studies have reported that glutamine, asparagine and leucine are essential for mTORC1 activation (37, 40, 41). Also, among ARSs, LARS has been identified to perform as an intracellular leucine sensor for mTORC1 signaling pathway (42). Enrichment in ARS activity in the gills of the susceptible family implies that mTORC1 signaling pathway is activated in the susceptible family, especially in response to VP_AHPND_ infection. Moreover, glutamine, supplying carbon and nitrogen, is a primary fuel for rapidly proliferating cells (43). It further suggests that cell proliferation and metabolism could influence susceptibility and resistance of shrimp against VP_AHPND_.

Ribosomes are essential ribonucleoprotein complexes that are the sites for protein synthesis in all cells (44). In the present study, ribosome was the significantly enriched GO term and KEGG pathway between the two families, especially at 6 h post-infection. Among the shared 1,124 DEGs, there were 20 ribosomal proteins (15 60S and 5 40S) upregulated in the resistant family compared with the susceptible family. These results are suggestive of increased ribosome biogenesis in the resistant family. It has been recognized that stress conditions like hypoxia and microbial infections can cause shortage of oxygen and increase reactive oxygen species (ROS), resulting in oxidative stress within the cells (45). As the key ROS targets, ribosomes could be damaged under oxidative damage, which cause translational errors and stop (46, 47). More recently, as suggested by Shcherbik and Pestov (48), low-level oxidative stress could lead to largely reversible modifications in rRNA and r-proteins, which could potentially promote selective translation of stress-response proteins and facilitate adaptive cellular responses. The differential expression of ribosomal protein genes between the two families suggests that ribosome biogenesis plays important roles in resistance or susceptibility to AHPND in *L. vannamei*. It appears that the elevated ribosome biogenesis could promote the gill cells of resistant family undergoing quick repair from oxidative damage induced by VP_AHPND_ infection.

### Differential cellular immune responses between the two families

Many studies with decapod crustaceans have suggested that gills are highly vulnerable to pathogenic infection (13, 16). Some inflammatory types, including bacterial granulomas, melanized nodule and hemocytic infiltration, were observed in the gills of infected shrimp (49, 50). In this study, transcription factors including HIF-1α, NF-κB factor Relish, and STAT, were mainly enriched at two time point’s comparison. HIF-1α, NF-κB and STAT have been reported to be major players in inflammation and their interactions could regulate the immune-metabolic response of the host cells during infection (51, 52). The expression of HIF-1α, Relish and Stat5b were significantly upregulated in the susceptible family at 0 and 6 h post-infection, demonstrating that these genes are potentially involved in defense and immune response of the susceptible family. It also suggests that some inflammatory responses might have occurred in the gill cells of susceptible shrimp. This speculation is further supported by the expression pattern of TNFRSF and LITAF, which has been reported to participate in the inflammation and immune responses against pathogens (53–55).

There is an important association between inflammation and hypoxia. For example, HIF-1α and NF-κB are two interdependent transcription factors that play important roles in the control of both inflammatory and hypoxic responses (56). The mTORC1, a positive regulator of HIF-1α expression and activity, could respond to hypoxia, and participate in immune cell activity during inflammation (57, 58). In shrimp, besides the inflammatory responses, *Vibrio* infection can cause the decrease of oxygen uptake during pathogenesis and induce hypoxia (59). Our results further indicate that HIF-1α could be involved in the response to VP_AHPND_-elicited hypoxia and the susceptible shrimp might be exposed to hypoxia condition compared to the resistant shrimp.

In the present study, we found that most immune DEGs including PRRs, antioxidant enzymes and proteins, and AMPs were highly expressed in the shrimp from the resistant family, suggesting the potential link between abundance of immune gene transcripts and AHPND resistance of *L. vannamei*. PRRs play crucial roles in the recognition of pathogen-associated molecular patterns (PAMPs) of invading microorganisms, and trigger subsequent innate immune responses (60). Among PRRs, C-type lectins are a large group of Ca^2+^-dependent carbohydrate-binding proteins, and have been reported to participate in immune recognition and phagocytosis through opsonization in crustaceans (61). The upregulation of C-type lectins in the gills of the resistant shrimp indicates that a rapid immune response could occur in the resistant shrimp during VP_AHPND_ infection. ROS can be scavenged by an antioxidant system involving antioxidant enzymes of glutathione-S-transferase (GST) and thioredoxin (Trx), and metal binding proteins ferritin and selenoprotein (62, 63). Our results showed these antioxidant enzymes and proteins were the most highly expressed in the resistant shrimp than the susceptible shrimp, suggesting the resistant shrimp have the strong capacity to reduce the extent of ROS-mediated damage especially during VP_AHPND_ infection. As effectors of immune response, shrimp AMPs, such as penaeidin (PEN), antilipopolysaccharide factor (ALF) and crustin, have been reported to kill or clean the infected pathogens directly (61). Consistent with a previously report in *L. stylirostris* (64), our findings also showed that the significant differences in the expression levels of some AMP transcripts were evidenced between the susceptible and resistant shrimp before and after *Vibrio* infection. Recent study demonstrated that macrophage-like phagocytes, highly expressing marker genes C-type lectins and ALFs, existed in shrimp hemolymph, which could effectively engulf VP (65). The higher expression of C-type lectins and ALFs in the gills of the resistant family might be related to the higher portion and activity of macrophage-like phagocytes in the resistant family. Taken together, our results might indicate that the basal immune response of the resistant shrimp is activated, which led to higher resistance of shrimp to disease.

## Conclusion

**Figure 8:**
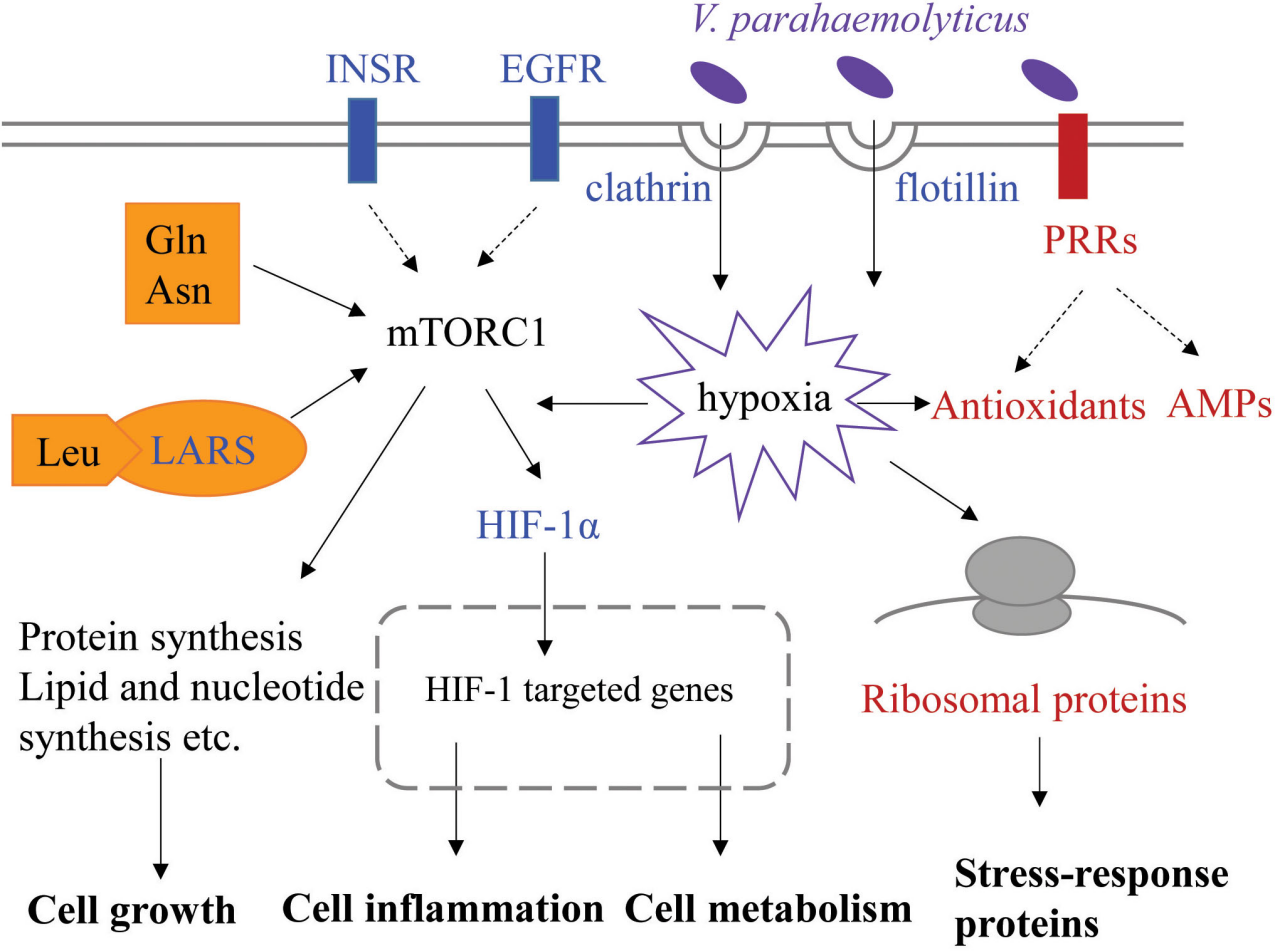
Putative key signaling pathways associated with resistance to VP_AHPND_ involved in *L. vannamei*. This represents the cellular process of gill cells at 0 and 6 h post-infection. The upregulated genes in the susceptible or resistant family are shown in blue or red font, respectively. INSR, insulin-like peptide receptor; EGFR, epidermal growth factor receptor; LARS, leucine-tRNA ligase; PRR, pattern recognition receptor; AMP, antimicrobial peptide.

Based on transcriptomic analysis of gill, we compared gene expression profiles of two families of *L. vannamei* differing in susceptibility to VP_AHPND_ both basally (before infection) and at 6 h post-infection. A total of 1,124 DEGs were identified between the resistant and susceptible families at two time points. The main finding of this study is that DEGs involved in endocytosis, protein synthesis and immune response could influence resistance of shrimp against VP_AHPND_ and all these processes could be related to mTORC1 signaling pathway. Here, we propose a theoretical model associated with resistance to VP_AHPND_ in *L. vannamei* (**Figure 8**). The susceptible shrimp have enhanced endocytosis mediated by receptors, clathrin or flotillin, which could provide a possible pathway of VP_AHPND_ entry into the gill cells. The invaded VP_AHPND_ could induce hypoxia and HIF-1α therefore accumulate and translocate to the nucleus, where it could interact with HIF-1β and promote transcription of its target genes. The higher expression of insulin-like peptide receptor, epidermal growth factor receptor and aminoacyl-tRNA synthetases could indicate the activated mTORC1 signaling pathway in the susceptible family, which lead to differences in cell growth and metabolism between the two families. However, in the resistant shrimp, the immune and metabolic homeostasis could not be easily disturbed during VP_AHPND_ infection. The upregulated PRRs could rapidly recognize PAMP and trigger various defense responses to clean the invaded VP_AHPND_. The elevated ribosome biogenesis and antioxidants expression could reduce the cellular damage and oxidative stress caused by VP_AHPND_-elicited hypoxia. Our data integrate the *Vibrio*-resistance phenotype and gene expression data, and provide new insights into the molecular mechanism for AHPND susceptibility or resistance.

## Data availability statement

The data presented in the study are deposited in the Sequence Read Archive (SRA) in NCBI, accession numbers SRR22936311-SRR22936318.

## Author contributions

FL designed the experiments. YL performed the experiments and data analysis. YL and FL wrote the manuscript. YY participated in sample collection. YY, SL and MS participated in data discussion and interpretation. All authors contributed to the article and approved the submitted version.
