# Supplementary material for: Comparative transcriptomic analysis of gill reveals genes belonging to mTORC1 signaling pathway associated with the resistance trait of shrimp to VPAHPND
Source: Front Immunol. 2023 Apr 18;14:1150628. doi: 10.3389/fimmu.2023.1150628 (PMC10151482; doi:10.3389/fimmu.2023.1150628)
Supplement: Supplementary file 1 [file DataSheet_1.docx]

Supplementary Material

**Supplementary Table S1** **Primer sequences and product size of target genes used for real-time PCR validation.**

| Gene | Gene ID | Forward primer (5′ to 3′) | Reverse primer (5′ to 3′) | Product size (bp) |
| --- | --- | --- | --- | --- |
| LARS | LVAN08417 | TGAAGGACACGGTGGAGTT | CTCTTCCTTGGTGCGTTCA | 134 |
| Rel | LVAN00031 | AGCCACCTGAGTGGAGATA | CCAAGAACTGTAAGGCACTC | 167 |
| Flo1 | LVAN15461 | GGAAATTCAGCGTCGTG | CACCTTGGGCATGGTAT | 283 |
| RhoA | LVAN12953 | GGTGATGGTGCCTGTGGTA | TAGTCCTCCTGACCTGCTGTG | 164 |
| rps21_1 | LVAN13157 | CATCATCTATGCCAAGGACC | CAAGCGAGCAAGACAATCA | 151 |
| rps21_2 | LVAN14425 | CATCATCTATGCCAAGGACC | CAAGCGAGCAAGACAATCA | 151 |
| ALF4 | LVAN20962 | CACTCGCCCTGATTGCTCT | GTGACCCATGAACTCCACCTC | 122 |
| HIF1-α | MSTRG.29494 | ACAGGCAGCCAAGGAACAC | GGAATCGACAGGTCAGGGT | 137 |
| WFDC5 | MSTRG.19928 | GGTGGCTGCAAAGGGAGTT | GCACGAGTGGTGGTAGTGGAT | 235 |
| rpl-37a | LVAN03363 | CTCAGGAATGCCATCGCTC | GTCGTCGTCACTGAGAAGGTC | 164 |
| ALF | LVAN12117 | TACTTCATCGGGTCCGTCAC | CGGTTTGGCTTCTTCTCG | 152 |
| CTL | LVAN05407 | CGGAGACGATCAGTGCA | CAGGTTGAGGCGGAGTT | 205 |
| peroxidase | MSTRG.10498 | TTCAGTCCGCAGTCCATCACAG | CGACACTCCGTTGCCATAGTCA | 297 |
| LITAF | MSTRG.39947 | GCAGTCAACGCACATGATCT | TTGTATTTGCCCAGGAAAGC | 210 |
| Insr | LVAN20732 | AAGTTAGTAACAGACGGCTCAC | AATCCCTGGAATGGATAGG | 289 |

**Supplementary Table S2 Summary statistics of Illumina RNA-seq data of *L. vannamei*.**

| Group | Raw reads | Clean reads | Clean bases (Gb) | Clean Q30 (%) | GC content (%) | Mapped reads ratio (%) | Detected total genes |
| --- | --- | --- | --- | --- | --- | --- | --- |
| S4383-0-G-1 | 78,516,062 | 78,107,180 | 11.66 | 93.53 | 49.56 | 84.77 | 19,488 |
| S4383-0-G-3 | 59,280,600 | 58,902,156 | 8.78 | 93.28 | 51.07 | 85.01 | 20,450 |
| S4383-6-G-1 | 53,645,964 | 53,300,030 | 7.96 | 92.81 | 49.08 | 83.43 | 18,900 |
| S4383-6-G-3 | 48,505,492 | 48,182,058 | 7.20 | 93.03 | 50.81 | 83.27 | 19,294 |
| R4345-0-G-1 | 58,369,948 | 58,087,232 | 8.67 | 93.21 | 50.04 | 85.28 | 18,726 |
| R4345-0-G-3 | 63,932,724 | 63,649,686 | 9.51 | 93.01 | 50.87 | 86.05 | 18,988 |
| R4345-6-G-1 | 47,695,886 | 47,551,722 | 7.11 | 93.64 | 50.63 | 86.04 | 17,823 |
| R4345-6-G-2 | 50,428,836 | 50,262,868 | 7.51 | 93.98 | 50.61 | 86.46 | 18,255 |

**Supplementary Table S3 Gene ontology (GO) enrichment analysis of differentially expressed genes (DEGs) in each comparison group S4383-0-G-vs-R4345-0-G or S4383-6-G-vs-R4345-6-G (*p* adjust < 0.05).** Biological process (BP), cellular component (CC), and molecular function (MF), respectively.

**Supplementary Table S4 KEGG pathway enrichment analysis of DEGs in each comparison group S4383-0-G-vs-R4345-0-G or S4383-6-G-vs-R4345-6-G (*P* < 0.05).**

**Supplementary Table S5 DEGs shared between the two comparison groups S4383-0-G vs R4345-0-G and S4383-6-G vs R4345-6-G.**

**Supplementary Table S6 GO enrichment analysis of DEGs shared between the two comparison groups S4383-0-G vs R4345-0-G and S4383-6-G vs R4345-6-G (*p* < 0.01).**

**Supplementary Table S7 KEGG enrichment analysis of DEGs shared between the two comparison groups S4383-0-G vs R4345-0-G and S4383-6-G vs R4345-6-G (*p* < 0.05).**


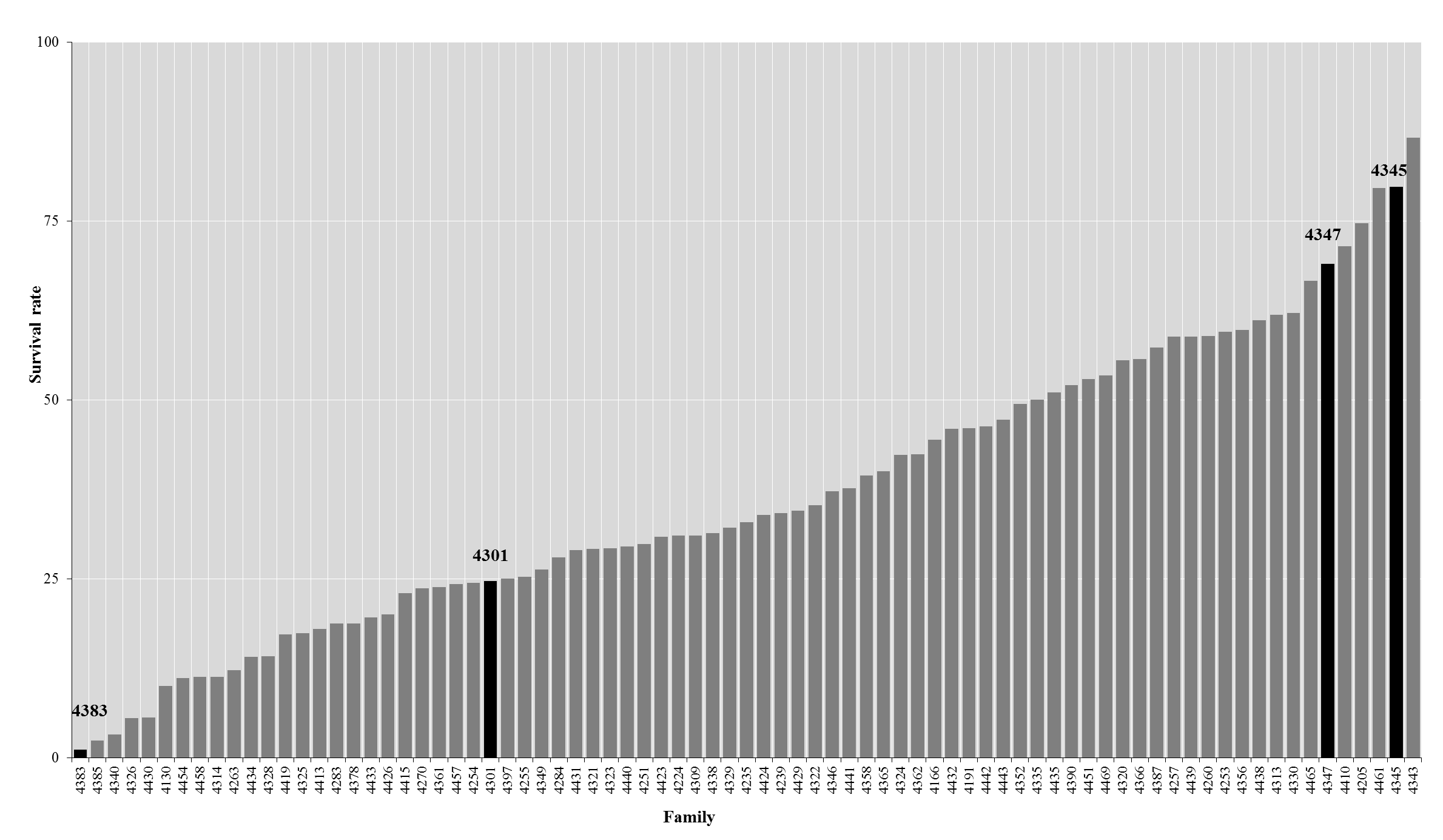


**Supplementary Figure S1.** Survival rate of *L. vannamei* families challenged by VP_AHPND_ in 2019.


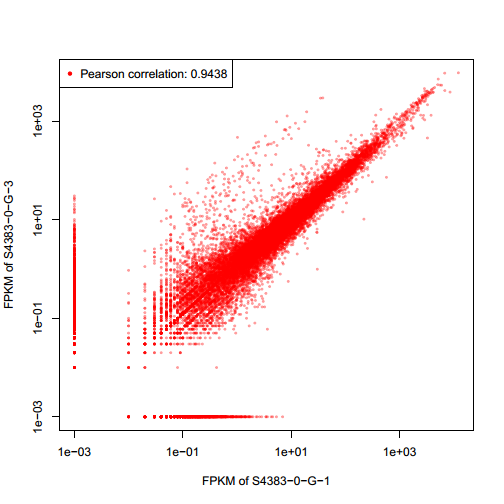

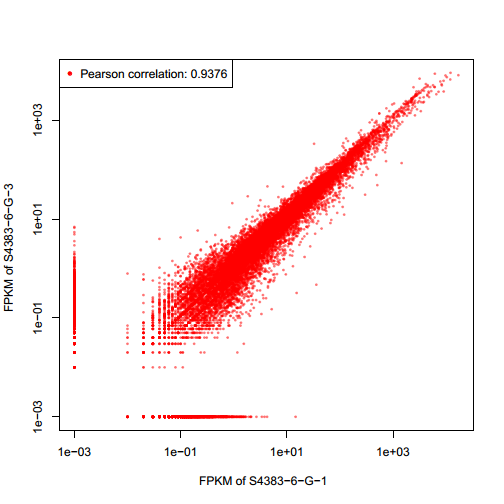


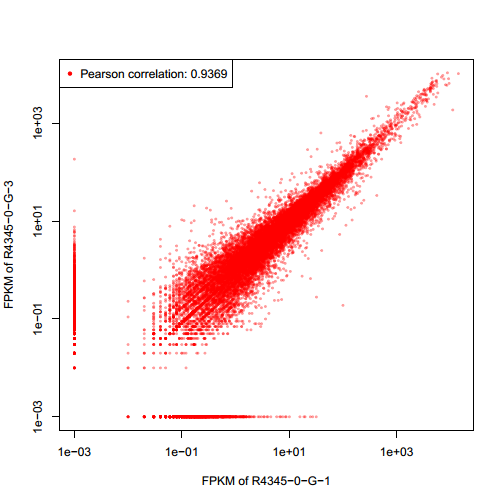

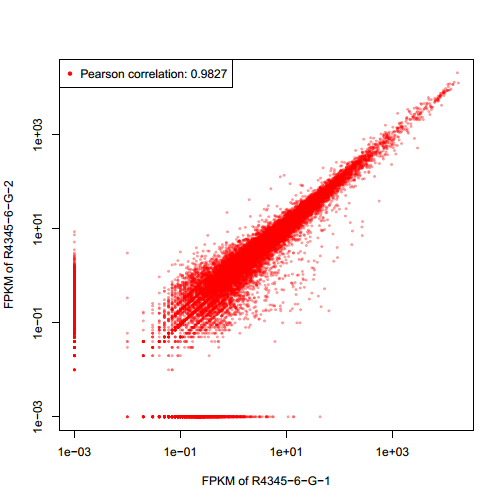


**Supplementary Figure S2.** Scatterplot matrix comparison of gene expression in two biological replicates of S4383 at 0 hpi, S4383 at 6 hpi, R4345 at 0 hpi and R4345 at 6 hpi.


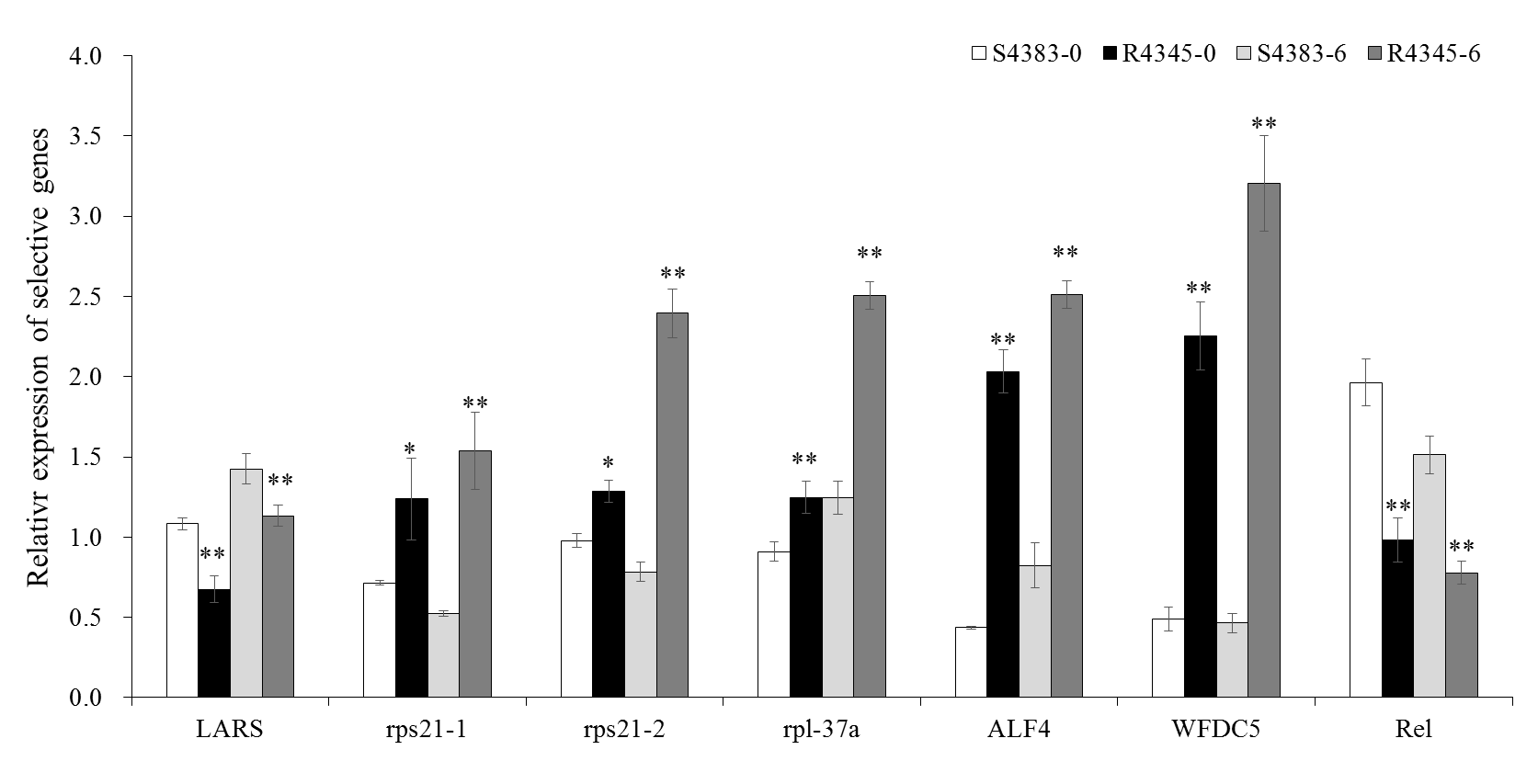


**Supplementary Figure S3.** qRT-PCR validation of selected DEGs identified by RNA-seq. Vertical bars represent the mean ± S.E. (n = 3). Significant differences between R4345 and S4383 at the same sampling point are indicated with an asterisk at *P* < 0.05, and two asterisks at *P* < 0.01.


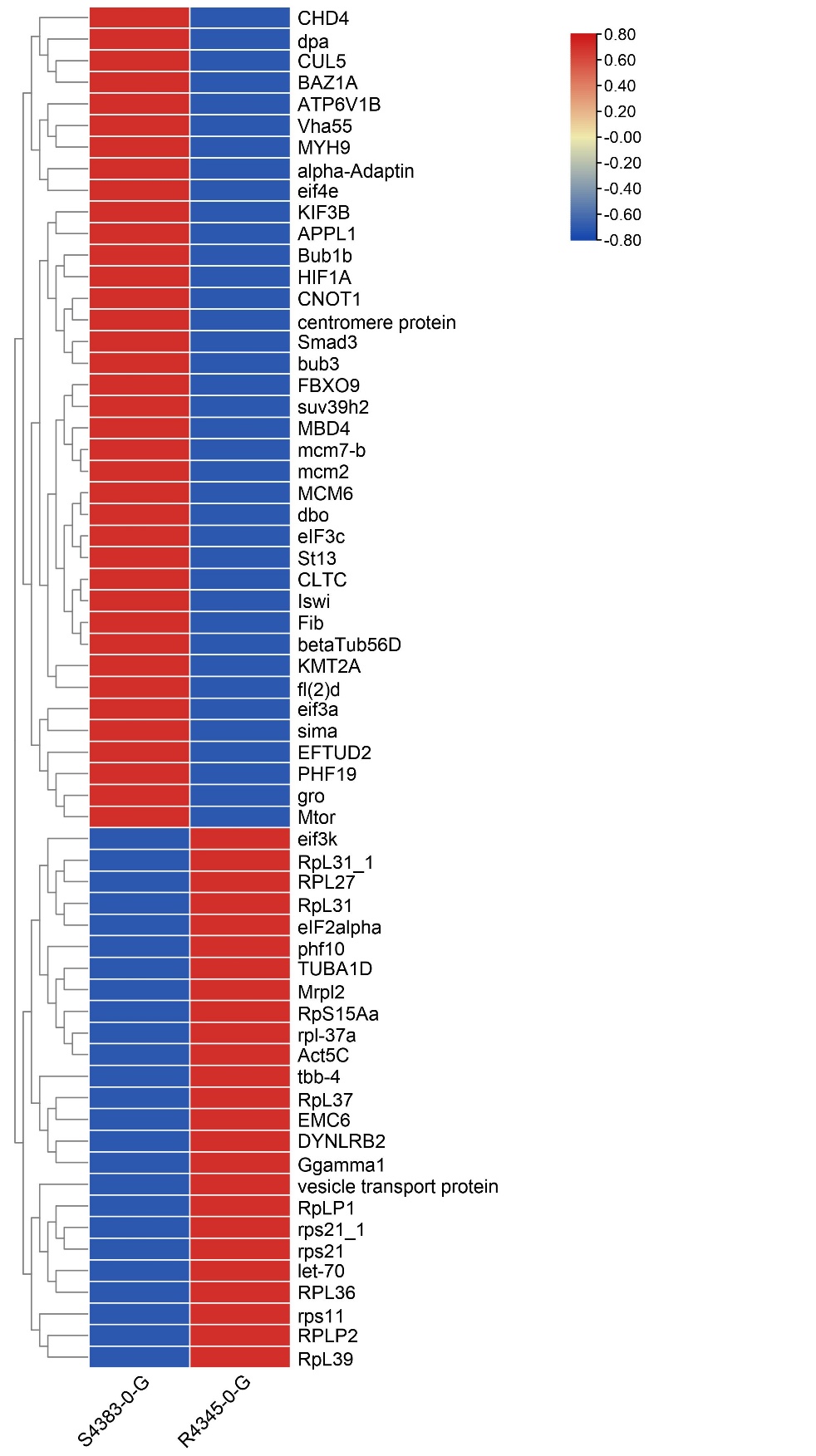


**Supplementary Figure S4.** Heatmap of the expression patterns of DEGs involved in macromolecular complex (GO:0032991) in S4383-0-G vs R4345-0-G.


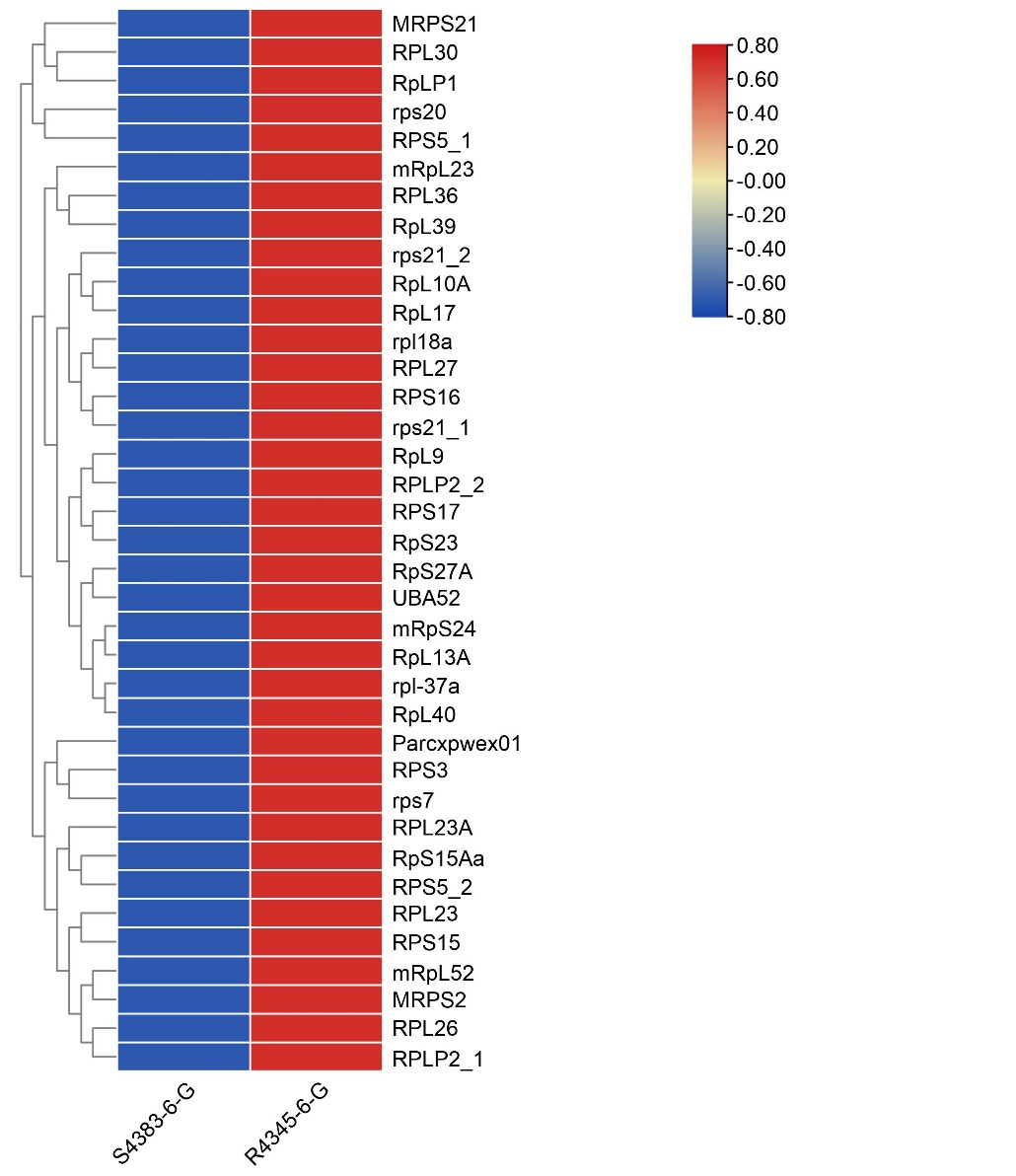


**Supplementary Figure S5.** Heatmap of the expression patterns of DEGs involved in ribosome (GO:0005840) in S4383-6-G vs R4345-6-G.


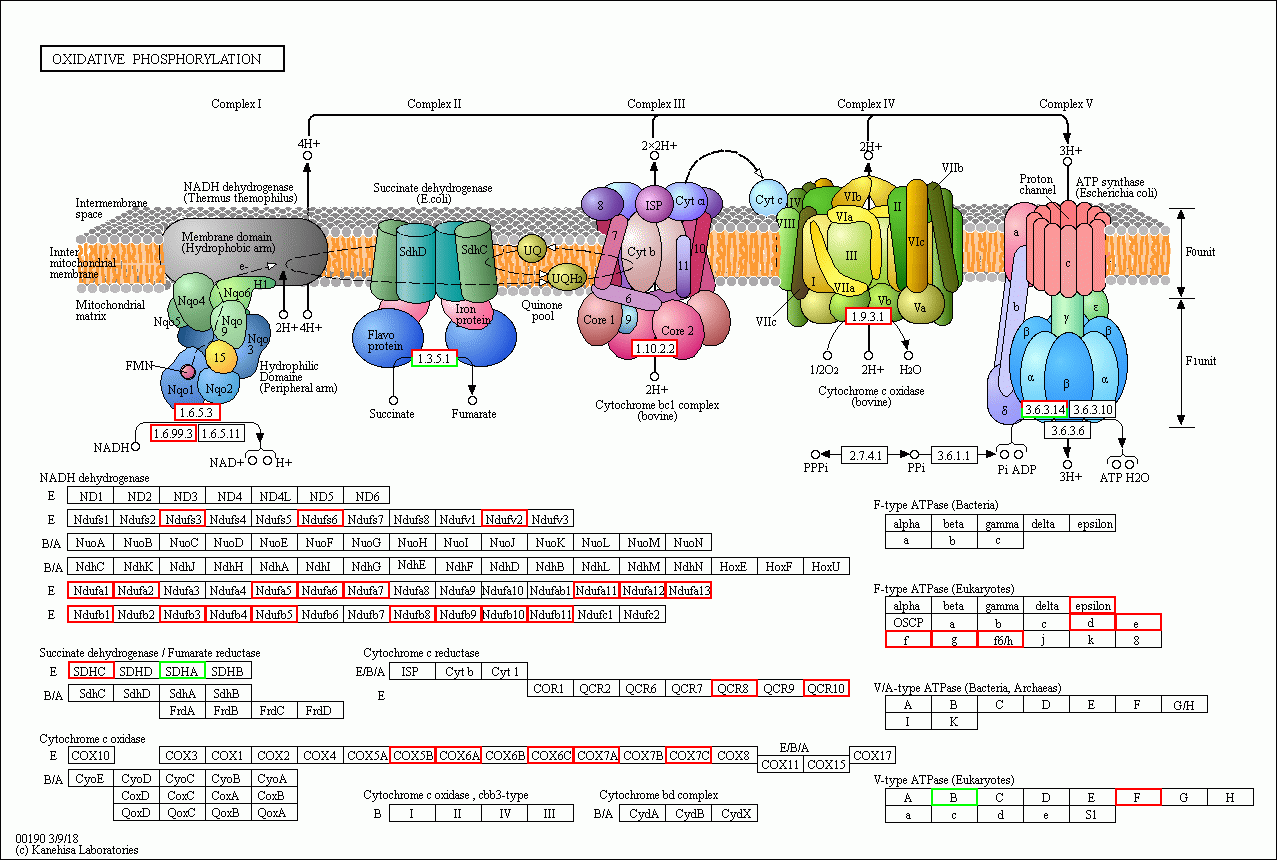


**Supplementary Figure S6. Expression pattern of DEGs involved in oxidative phosphorylation (map00190) between S4383-6-G and R4345-6-G.** The pathway is based on KEGG pathway analysis. The up-regulated genes in S4383-6-G and R4345-6-G are boxed by green and red, respectively.

**A**


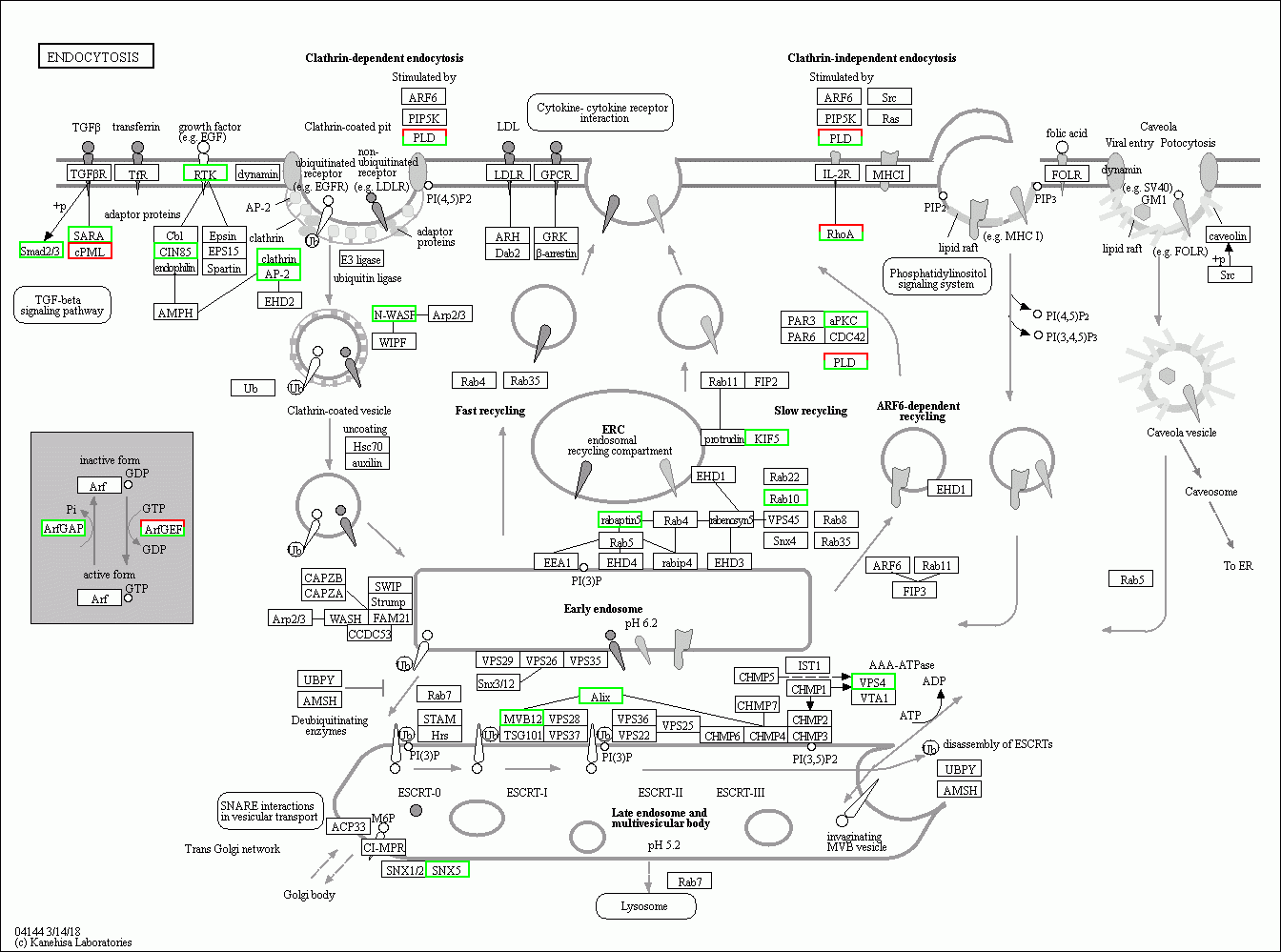


**B**


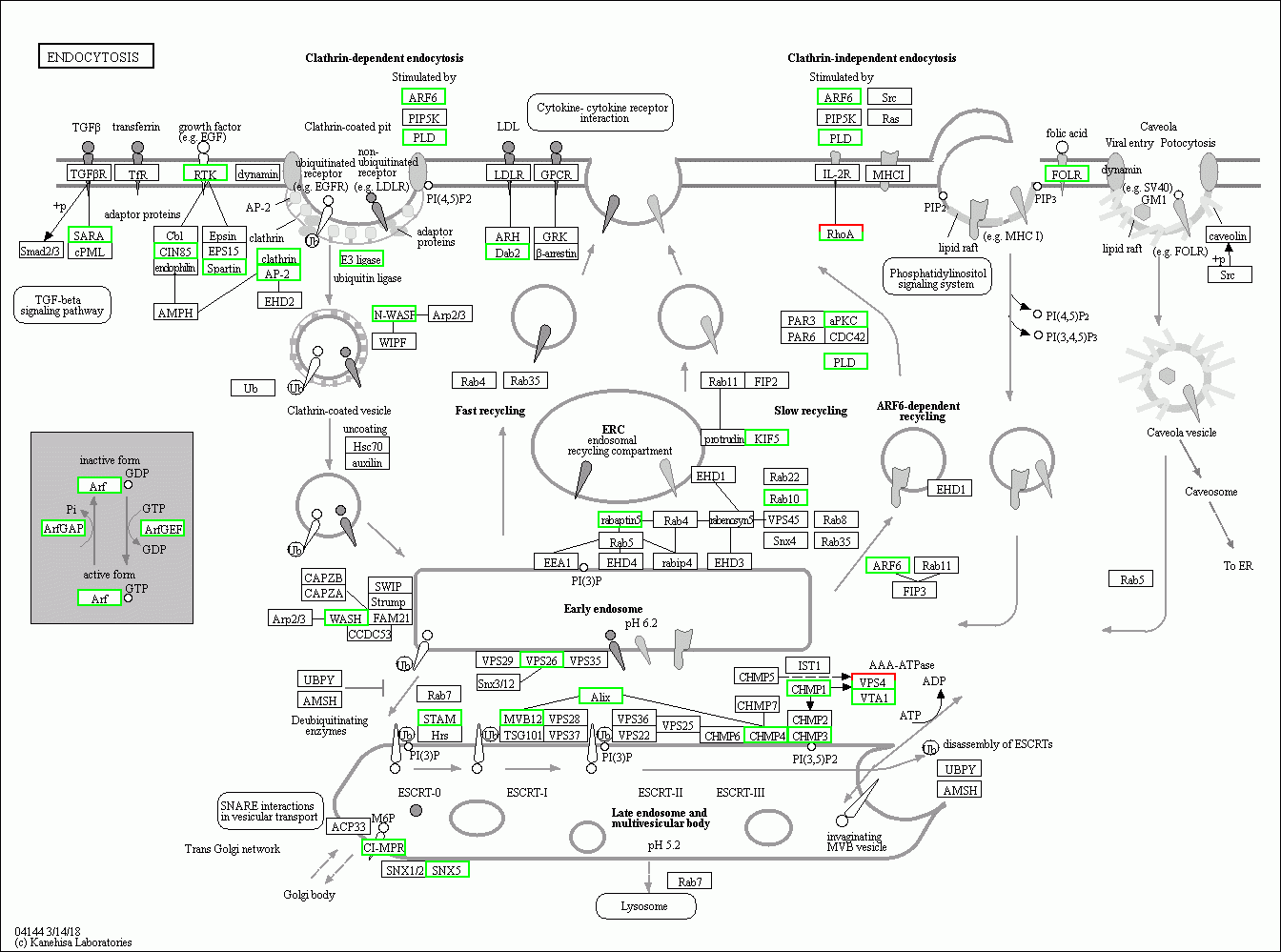


**Supplementary Figure S7. Expression pattern of DEGs involved in endocytosis (map04144) between (A) S4383-0-G vs R4345-0-G, (B) S4383-6-G vs R4345-6-G.** The pathway is based on KEGG pathway analysis. The up-regulated genes in S4383 and R4345 are boxed by green and red, respectively.

**A**


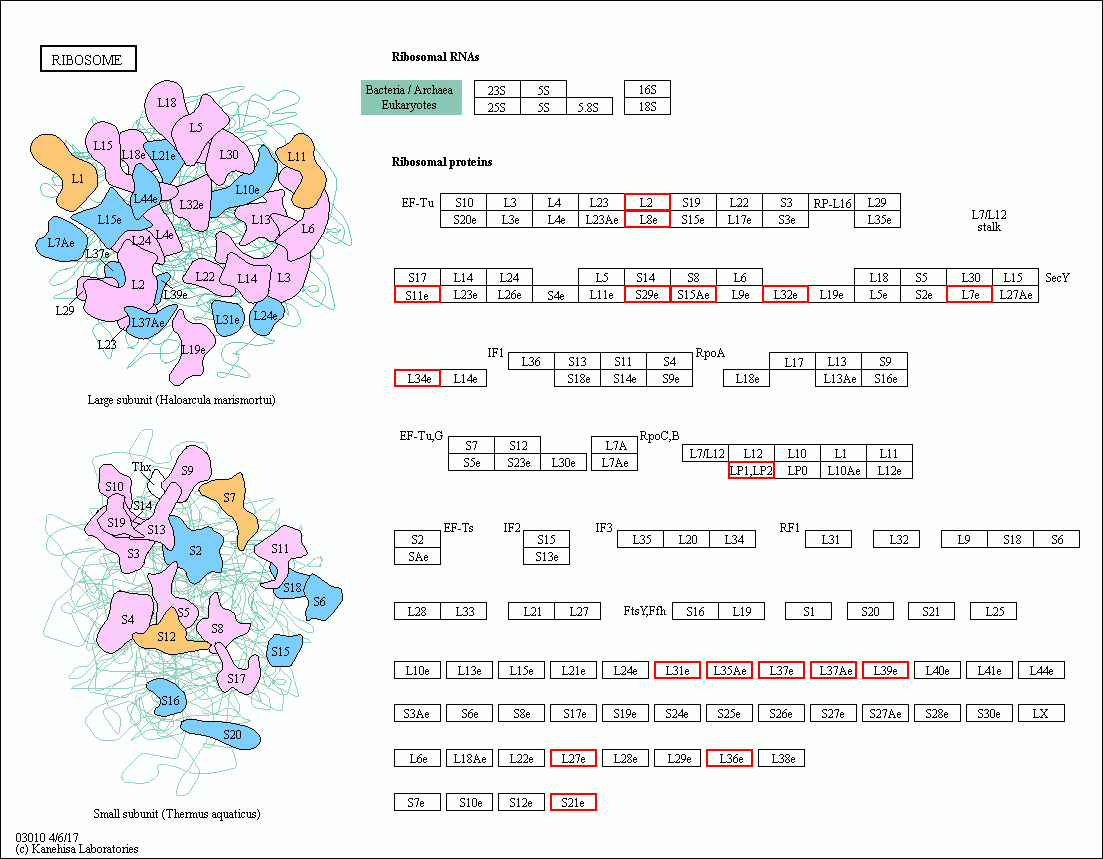


**B**


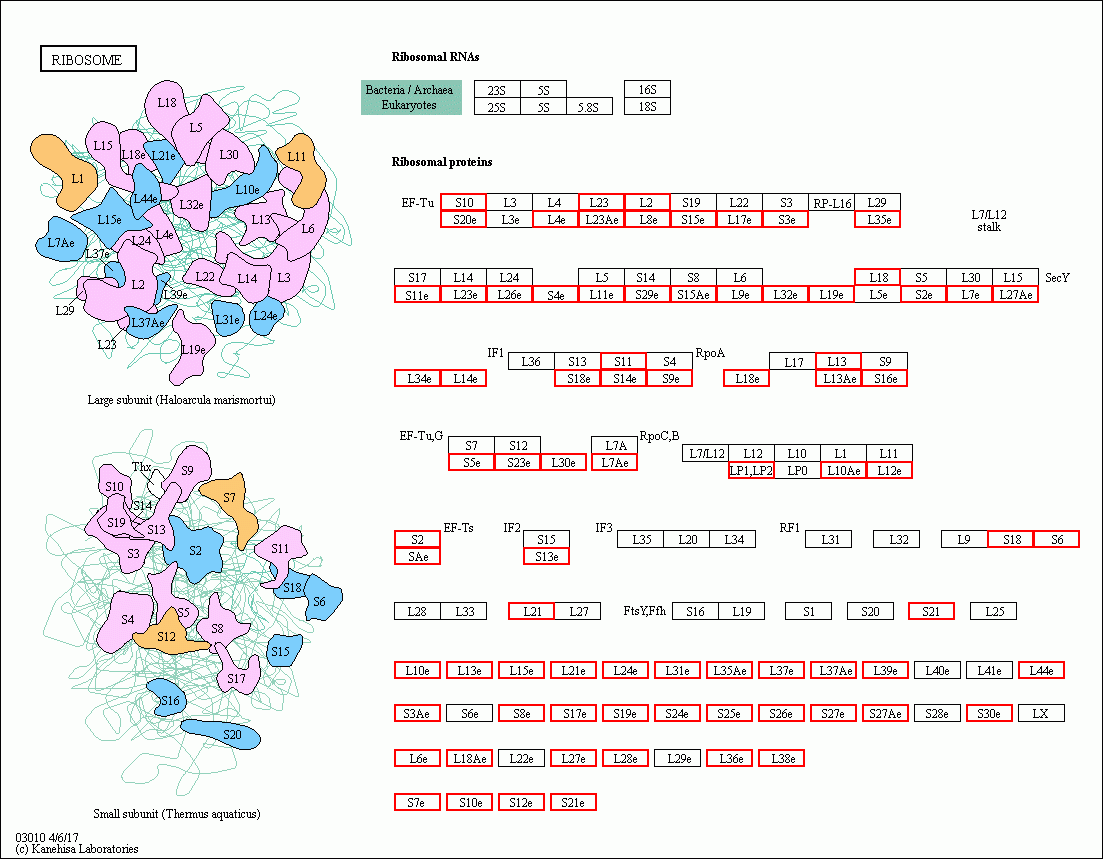


**Supplementary Figure S8. Expression pattern of DEGs involved in ribosome (map03010) between (A) S4383-0-G vs R4345-0-G, (B) S4383-6-G vs R4345-6-G.** The pathway is based on KEGG pathway analysis. The upregulated genes in R4345-6-G are boxed by red.


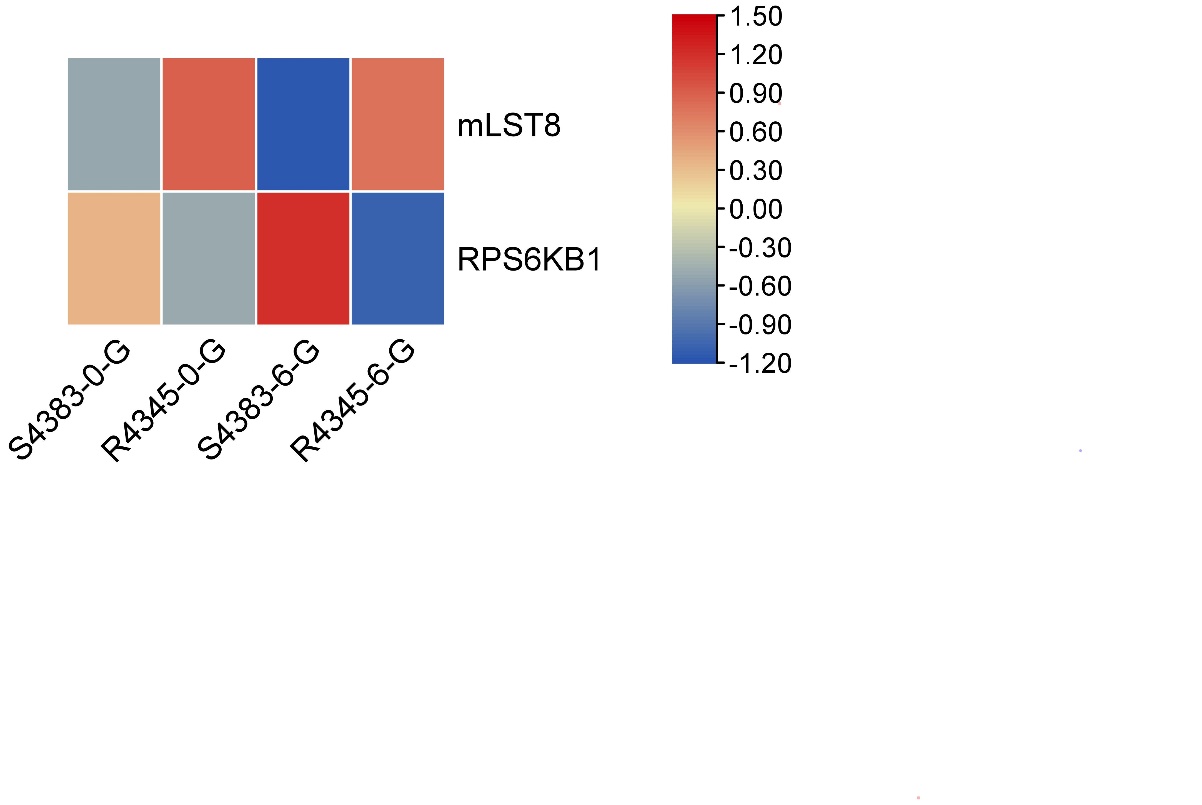


**Supplementary Figure S9. Heatmap of the expression pattern of mLST8 and RPS6KB1 in gills of S4383 and R4345.**

**A**


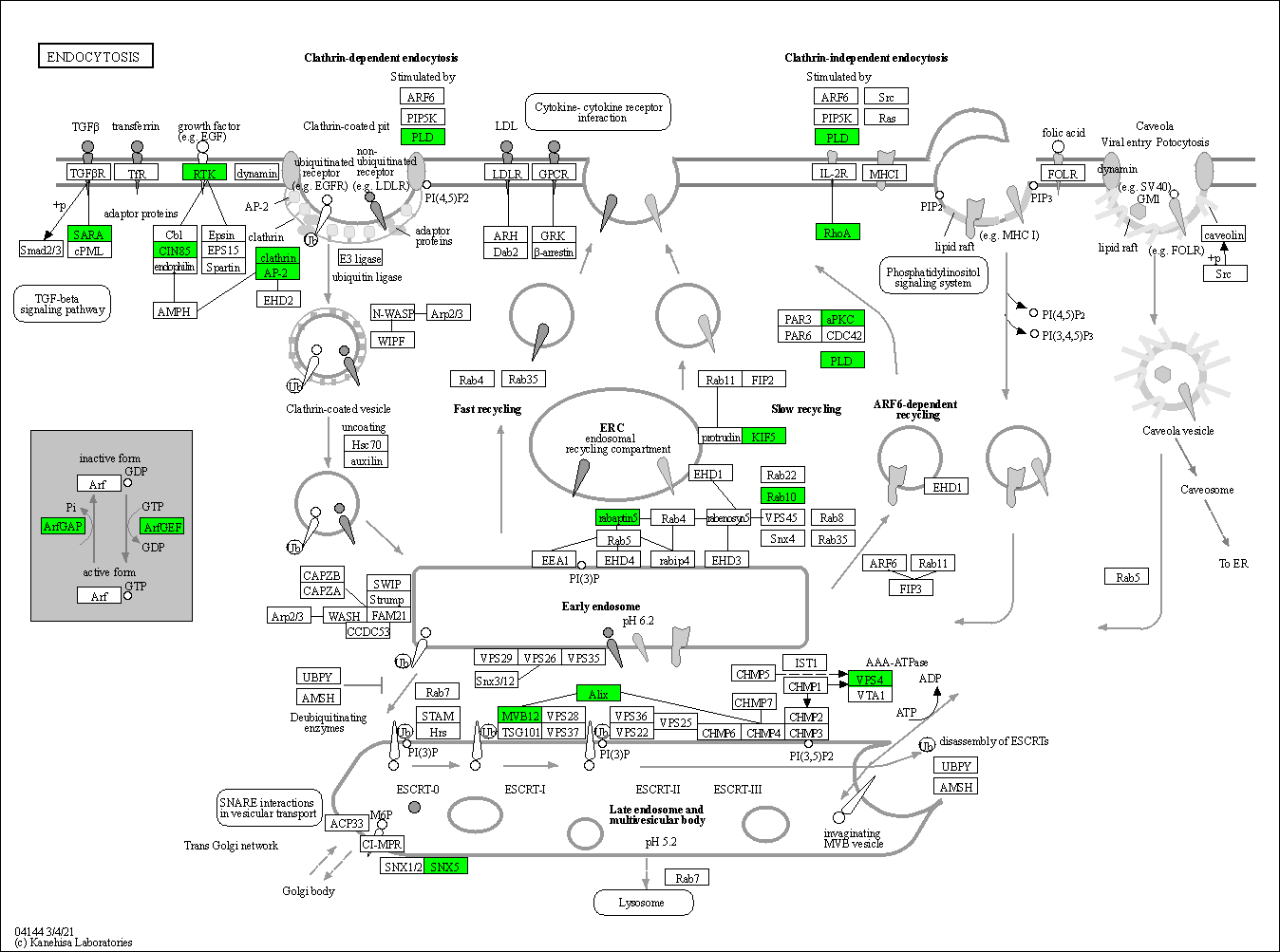


**B**


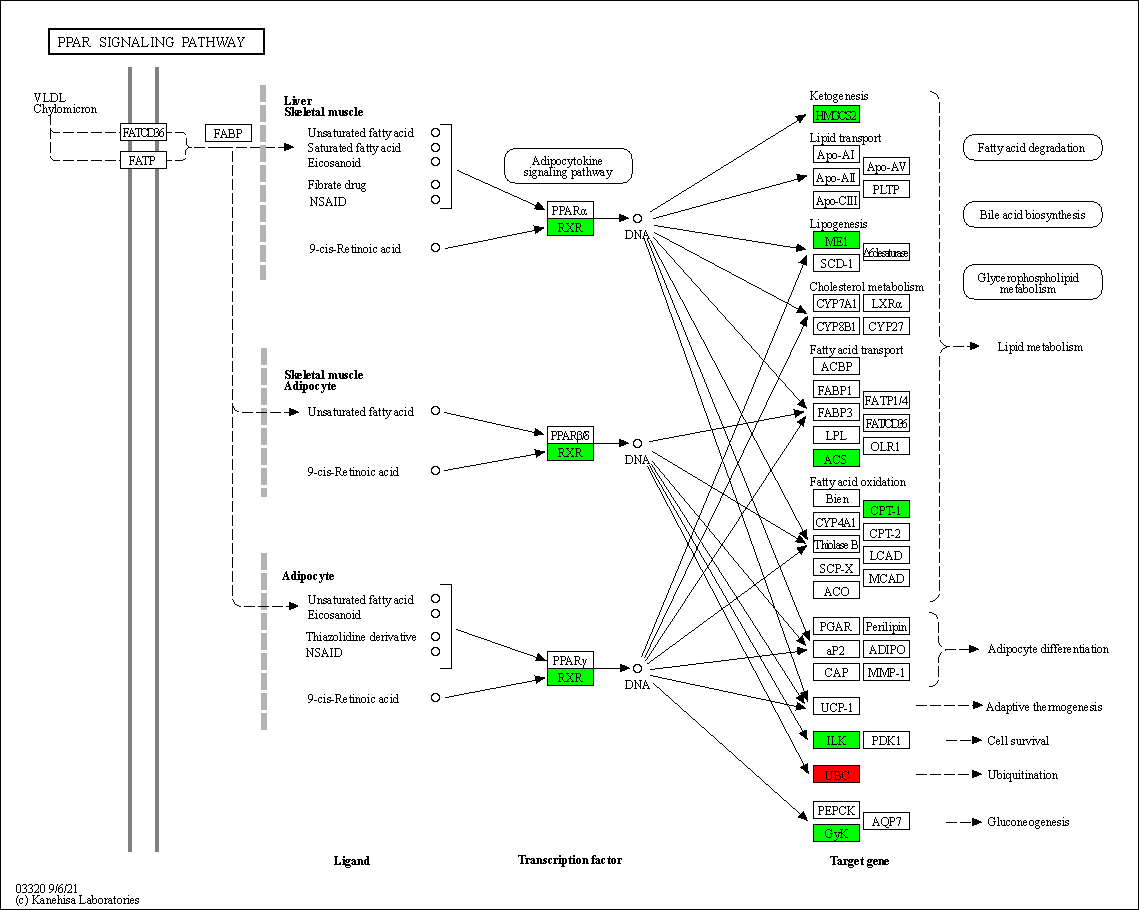


**Supplementary Figure S10. Expression pattern of DEGs shared between the two comparison groups S4383-0-G vs R4345-0-G and S4383-6-G vs R4345-6-G involved in (A) endocytosis (map04144) and (B) PPAR signaling pathway (map03320).** The pathway is based on KEGG pathway analysis. The upregulated genes in S4383-0-G and S4383-6-G are marked by green and the upregulated genes in R4345-0-G and R4345-6-G are marked by red.
